# Supplementary material for: Molecular engineering of dihydroxyanthraquinone-based electrolytes for high-capacity aqueous organic redox flow batteries
Source: Nat Commun. 2022 Aug 12;13:4746. doi: 10.1038/s41467-022-32424-8 (PMC9374662; doi:10.1038/s41467-022-32424-8)
Supplement: Supplementary file 1 — Supplementary Information [file 41467_2022_32424_MOESM1_ESM.pdf]

Supplementary Information

Molecular engineering of dihydroxyanthraquinone-based electrolytes for high-capacity aqueous organic redox flow batteries

Shiqiang Huang[1,3], Hang Zhang[1,3], Manohar Salla[1], Jiahao Zhuang[1], Yongfeng Zhi[1], Xun Wang[1], Qing Wang[1,2] *

[1]Department of Materials Science and Engineering, National University of Singapore, Singapore 117575, Singapore

[2] National University of Singapore (Suzhou) Research Institute, 377 Lin Quan Street, Suzhou Industrial Park, Suzhou, Jiangsu, 215123 P. R. China

[3]These authors contributed equally: Shiqiang Huang, Hang Zhang

*e-mail: msewq@nus.edu.sg

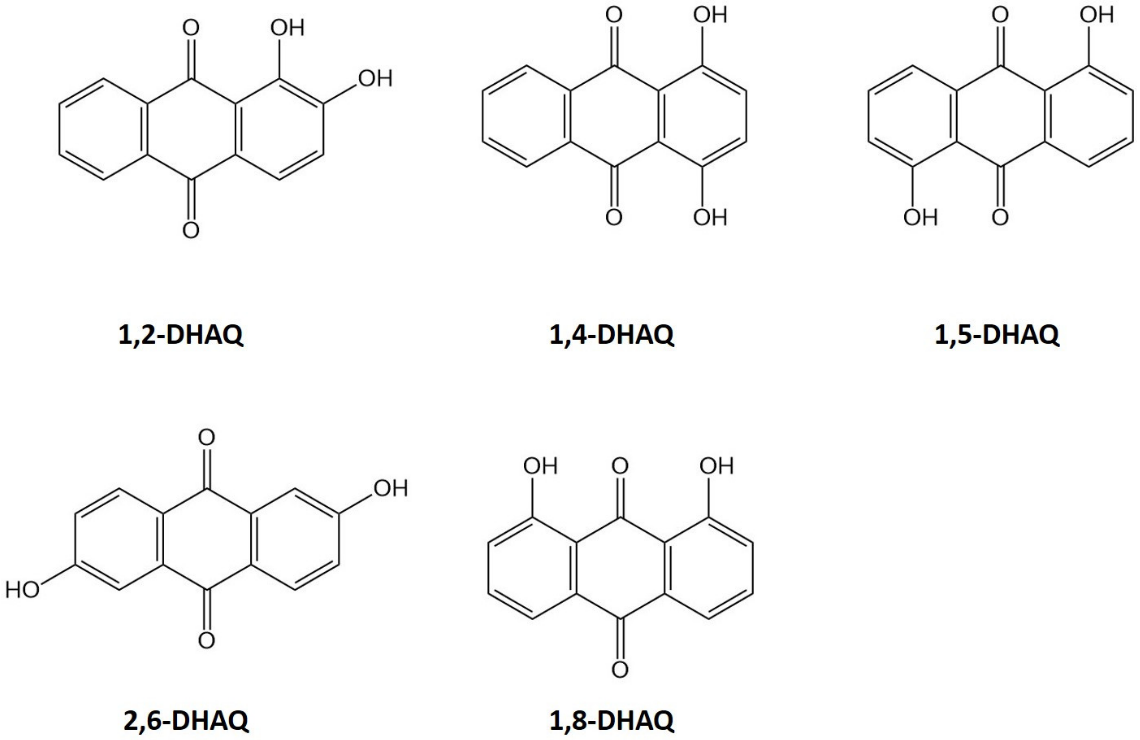

Supplementary Figure 1. Molecular structures of the studied DHAQs isomers.

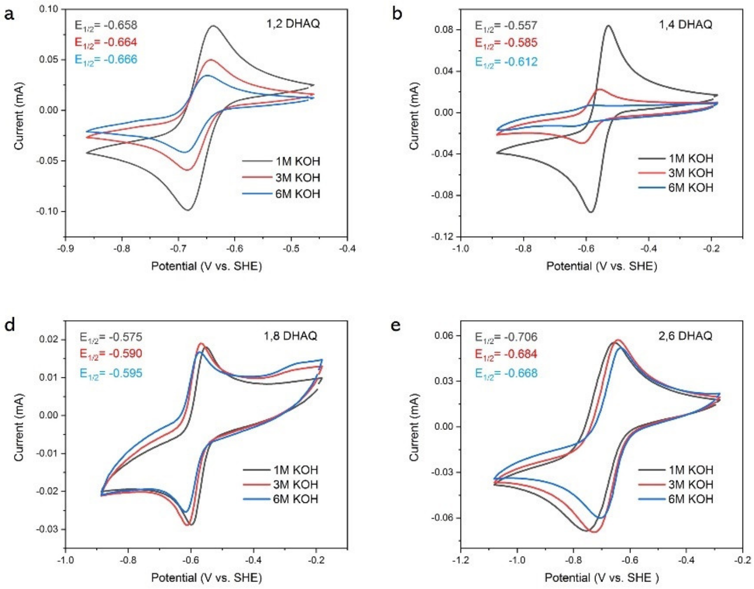

Supplementary Figure 2. CV tests of DHAQs. a, 1,2-DHAQ. b, 1,4-DHAQ. c, 1,5-DHAQ. d, 1,8-DHAQ and e, 2,6-DHAQ in 1 M KOH, 3 M KOH and 6 M KOH solution. The scan rate was 50 mV/s. All tests were conducted at 25 ±1 °C

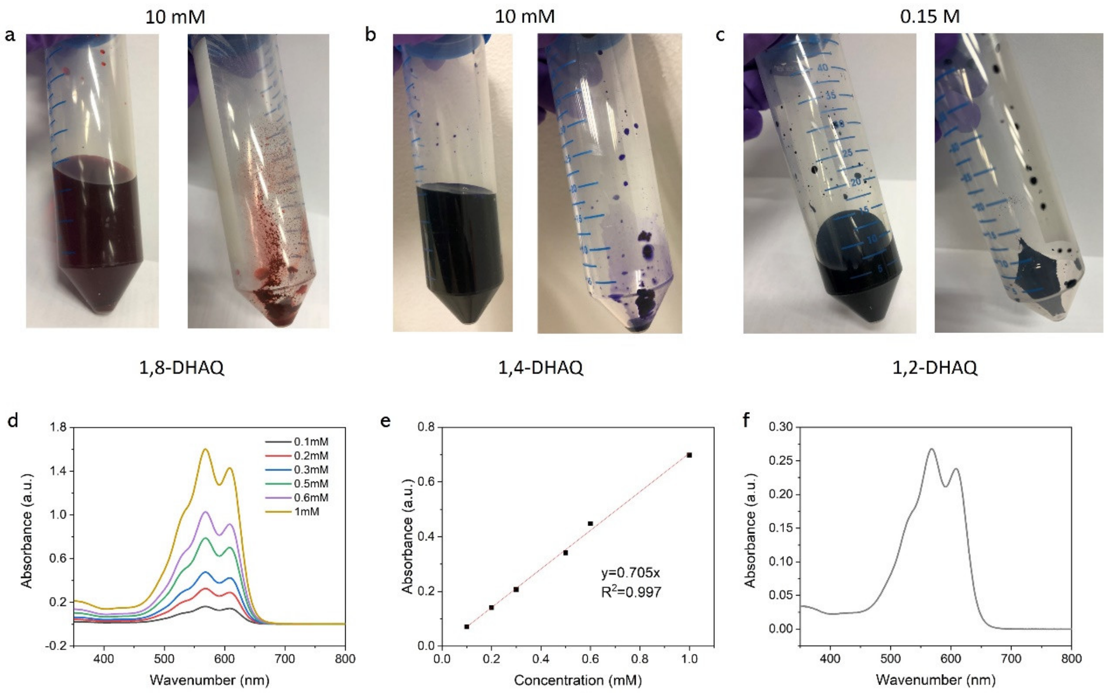

Supplementary Figure 3. Solubility test of DHAQs. a, 1.8-DHAQ. b,1,4-DHAQ and c, 1,2-DHAQ in 1 M KOH solution. It was tested by dissolving DHAQs into 1 M KOH solution until reaching saturated state. For 1,8-DHAQ and 1,4-DHAQ, the solubility is less than 10 mM in 1 M KOH, and for 1,2-DHAQ, the solubility is around 0.15 M in 1 M KOH. The solubility of 1,2-DHAQ was further evaluated by UV-Vis spectrophotometry. The solubility limit of 1,2-DHAQ was measured by adding the 1,2-DHAQ into 1 M KOH until no further solid could be dissolved. And then the solution was centrifuged and the saturated solution of 1,2-DHAQ was obtained, which was further filtrated and diluted for 1000 times for UV-Vis test. d and e, The pre-calibrated absorbance concentration curve of known concentrations of 1,2-DHAQ. f, The saturated concentration of 1,2-DHAQ (calculated to be 0.162 M). As a result, these two methods matches well and the further solubility of 1,5-DHAQ and 2,6-DHAQ were tested by dissolving DHAQs into solution until reaching saturated state.

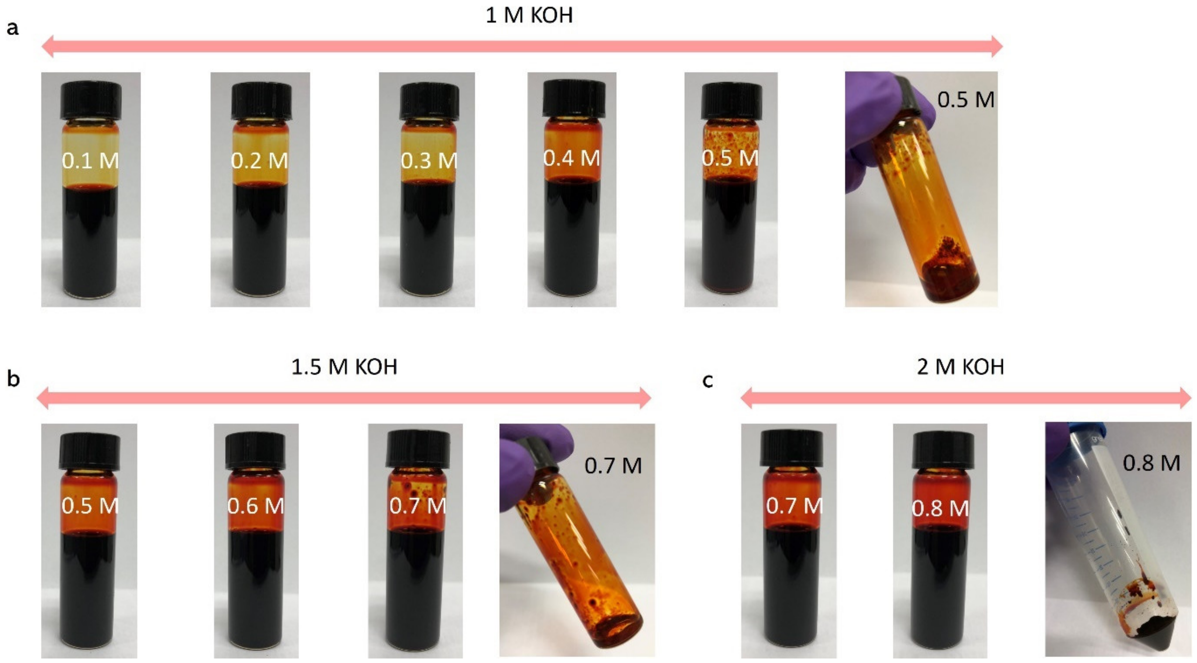

Supplementary Figure 4. Solubility test of 2,6-DHAQ in KOH solutions. a, The solubility test of 2,6-DHAQ in 1 M KOH solution, it was 0.5 M. b, The solubility test of 2,6-DHAQ in 1.5 M KOH solution, it was 0.7 M. c, The solubility test of 2,6-DHAQ in 1 M KOH solution, it was 0.8 M. The solubility was tested by dissolving 2,6-DHAQ into KOH solutions until reaching saturated state at 25 ±1°C.

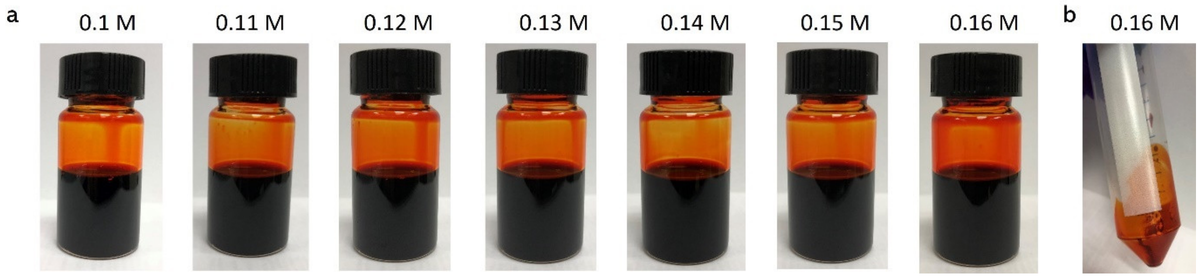

Supplementary Figure 5. Solubility of 1,5-DHAQ in 1 M KOH solution. a, Dissolving 1,5-DHAQ into 1 M KOH solution until reaching saturated state. b, Undissolved 1,5-DHAQ was observed after centrifuge when reach the saturated state of 1.6 M.

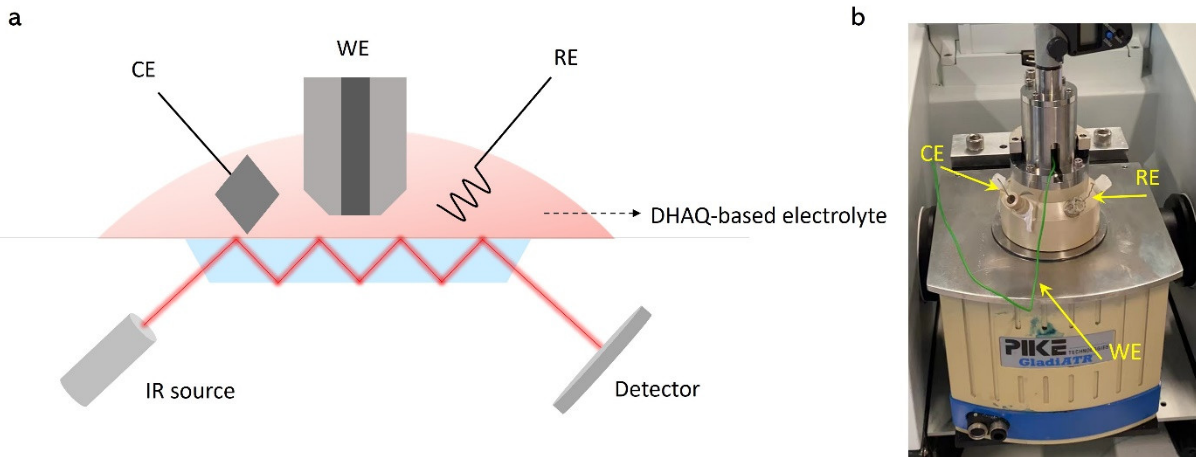

Supplementary Figure 6. Operando ATR-FTIR setup for 1,5-DHAQ and 2,6-DHAQ. a, Schematic of the setup. b, The photographic picture of the cell schematic.

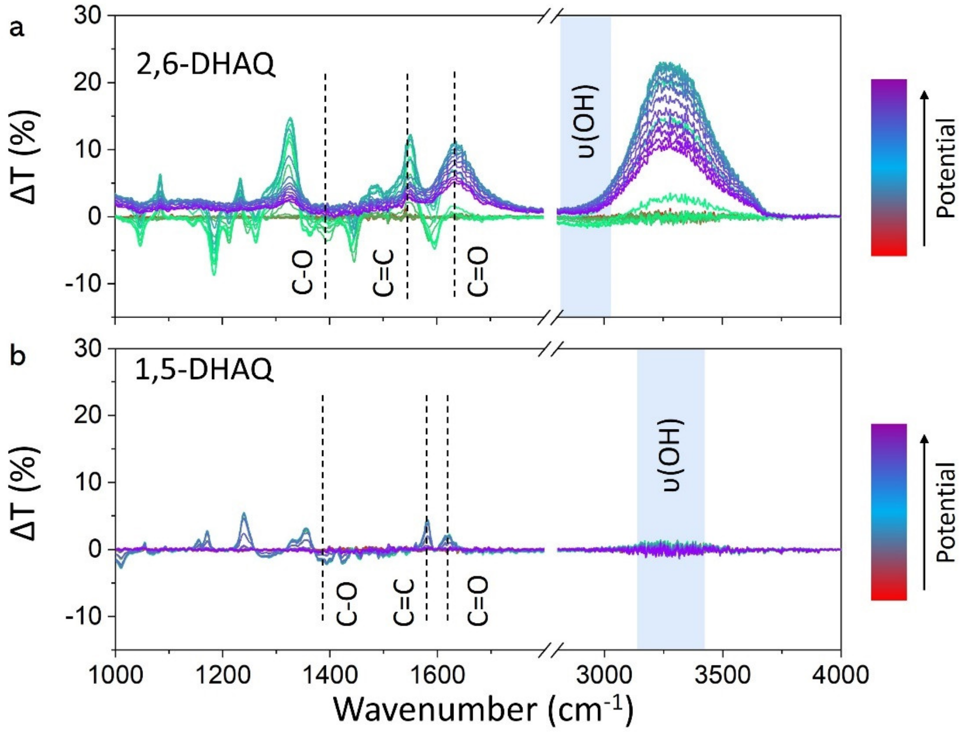

Supplementary Figure 7. Operando FTIR tests of 2,6-DHAQ and 1,5-DHAQ. Evolution of the FTIR spectra of a, 2,6-DHAQ and b, 1,5-DHAQ during reduction and oxidation processes in the first cycle of CV scan. ΔT is the transmittance difference of the sample relative to that before CV scan.

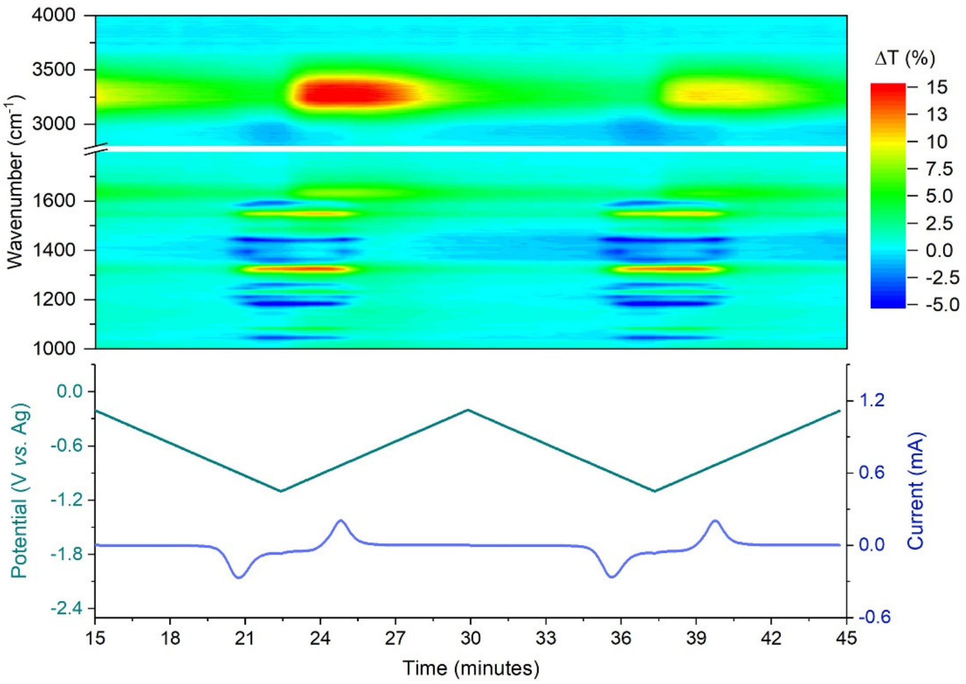

Supplementary Figure 8. Operando FTIR spectra of 2,6-DHAQ during the second and third CV scans. ΔT is the transmittance difference of the sample relative to that before CV scan. The tests were conducted at 25 ±1 °C.

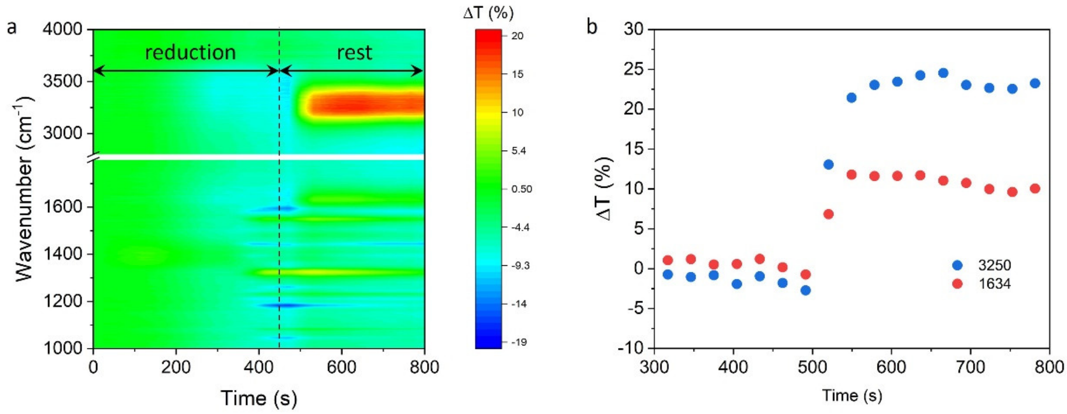

Supplementary Figure 9. a, Evolution of the operando FTIR spectra of 2,6-DHAQ during reduction and resting processes. b, ΔT vs. time plot at 1634 cm^{-1} and 3250 cm^{-1}. ΔT is the transmittance difference of the sample relative to that before CV scan. The test was conducted at 25 ±1 °C.

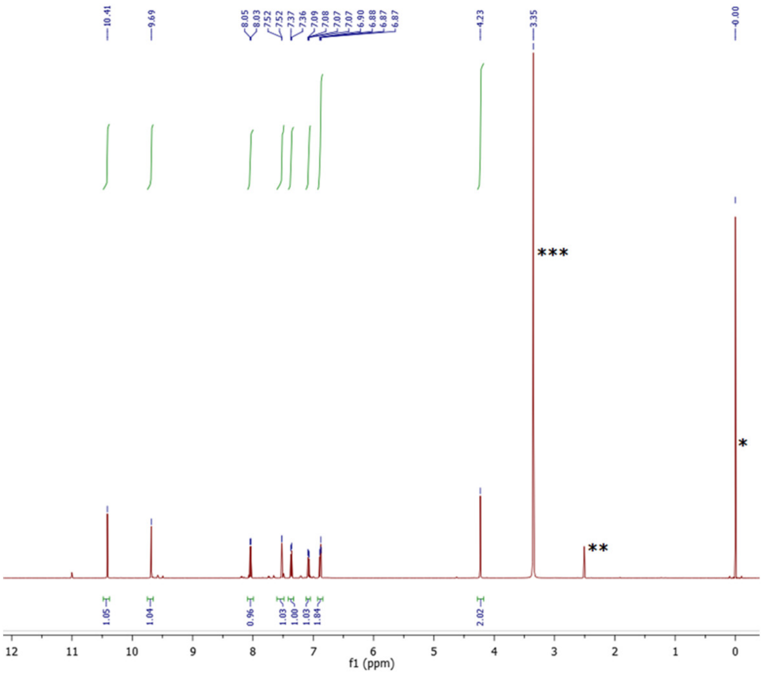

Supplementary Figure 10. ^{1}H NMR spectrum of 2,6-DHA in DMSO-d_6. * Trimethylsilane (TMS) reference peak, ** Residual DMSO in DMSO-d_6, *** Residual H_2O in DMSO-d_6. The synthesis of 2.6-DHA was carried out by following the reported procedure.[1]

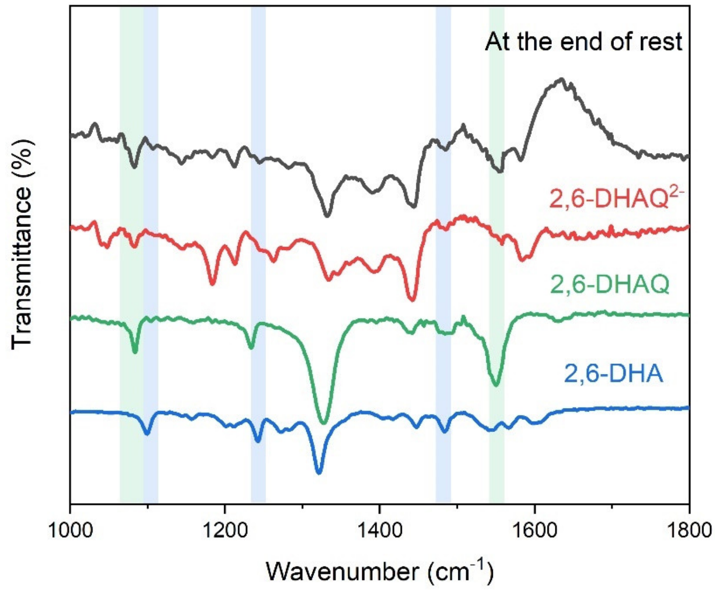

Supplementary Figure 11. Comparison of 2,6-DHAQ-based compounds. FTIR spectrum of the electrolyte at the end of resting, and the spectra of 2,6-DHAQ^{2-}, 2,6-DHAQ and 2,6-DHA. All tests were conducted at 25 ±1 °C.

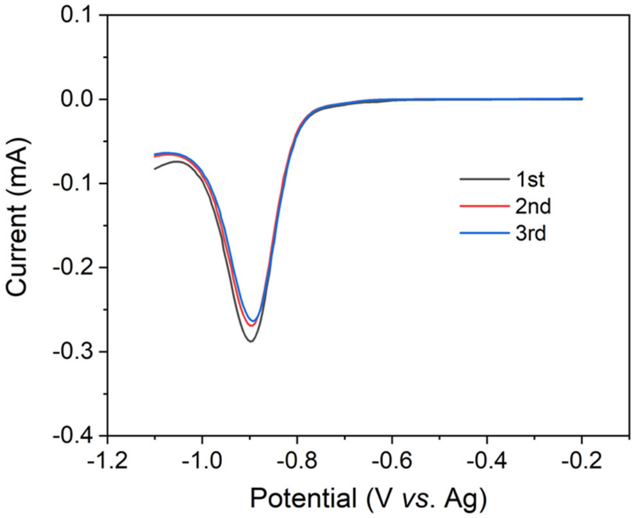

Supplementary Figure 12. Comparison of reduction currents of 2,6-DHAQ during operando FTIR tests. Replot of the reduction current of 2,6-DHAQ during the three consecutive CV scans of FTIR testing. Replot means the currents were overlapped by three reduction process of operando FTIR tests in Supplementary Figures 7 and 8, which were conducted at 25 ±1 °C.

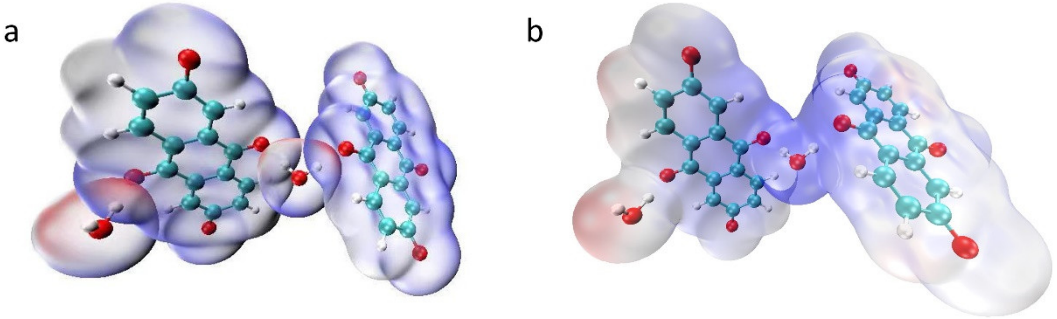

Supplementary Figure 13. The hydrogen bond formation analysis. Surface electrostatic potential maps of a, four individual molecules (two 2,6-DHAQ^{2-} and two H_2O) and b, 2(2,6-DHAQ^{2-})·2H_2O complex.

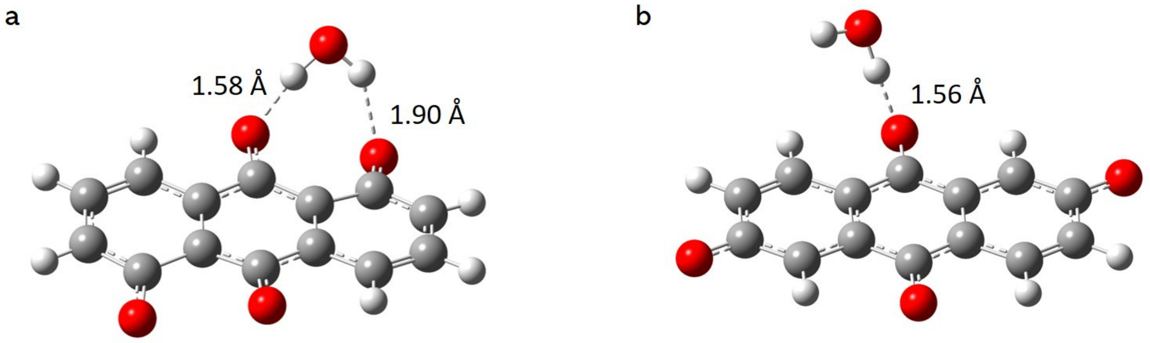

Supplementary Figure 14. The strength analysis of hydrogen bond. Optimized structures of (a) 1,5-DHAQ^{2-}·H_2O and (b) 2,6-DHAQ^{2-}·H_2O. Red ball: O; Grey ball: C; white ball: H.

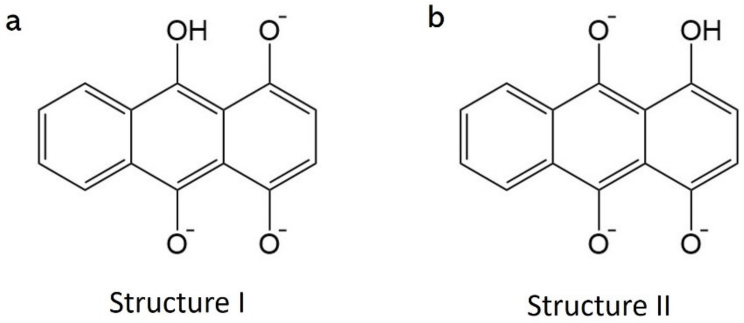

Supplementary Figure 15. Possible protonation structures of reduced 1,5-DHAQ. a, The protonation of the middle -O$^-$ group. b, The protonation of the side -O$^-$ group.

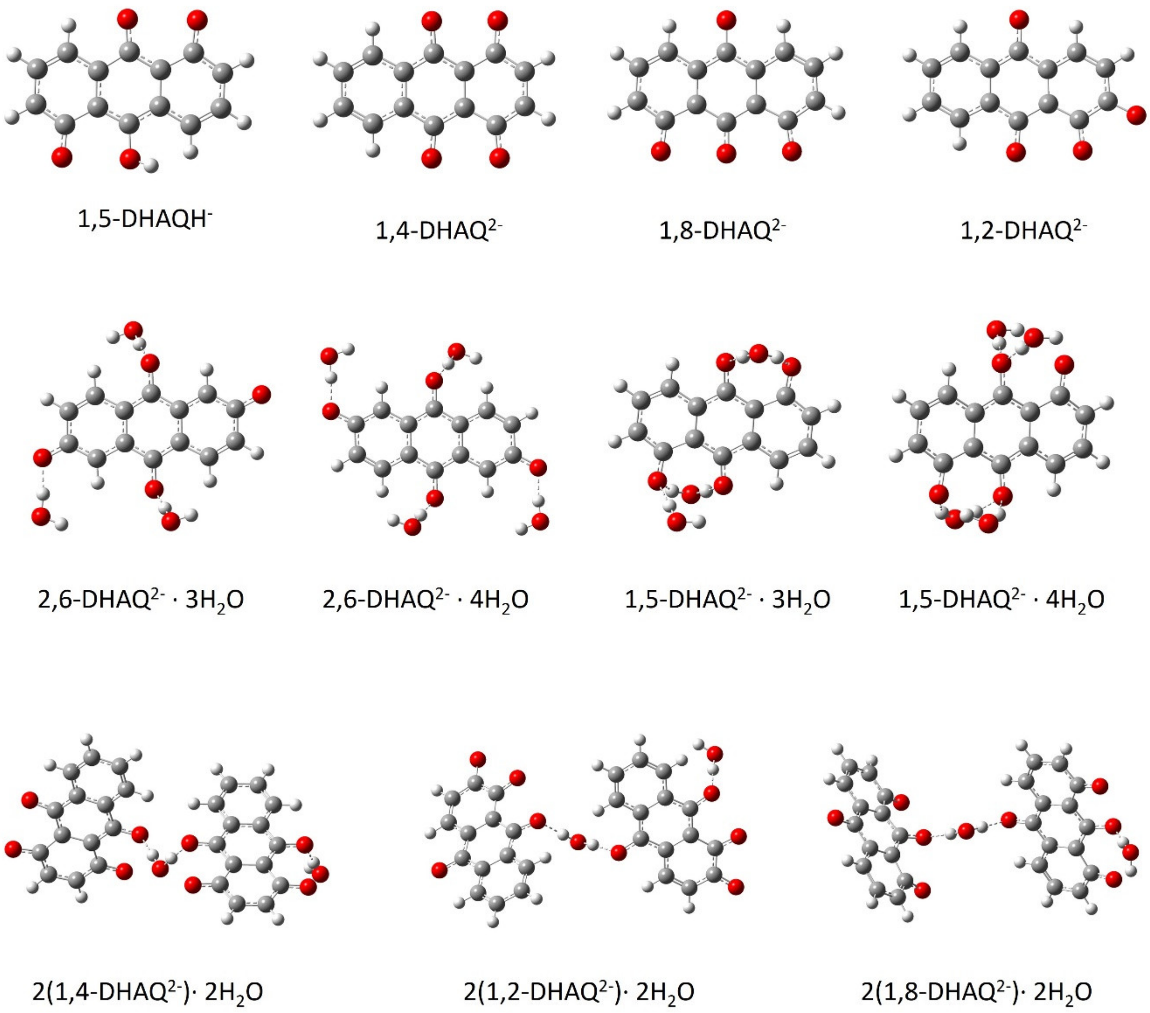

Supplementary Figure 16. Optimized structures of DHAQH$^-$, DHAQ^{2-}, DHAQ^{2-} · nH_2O and 2DHAQ^{2-}·2H_2O. Red ball: O; Grey ball: C; white ball: H.

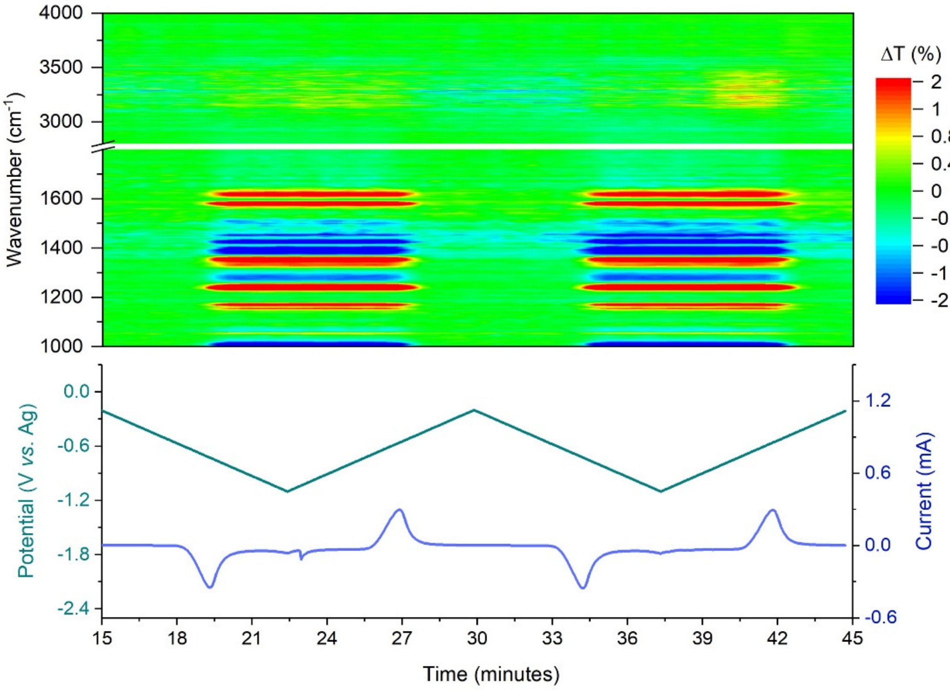

Supplementary Figure 17. Operando FTIR spectra of 1,5-DHAQ during the second and third CV scans. ΔT is the transmittance difference of the sample relative to that before CV scan. All the tests were conducted at 25 ±1 °C.

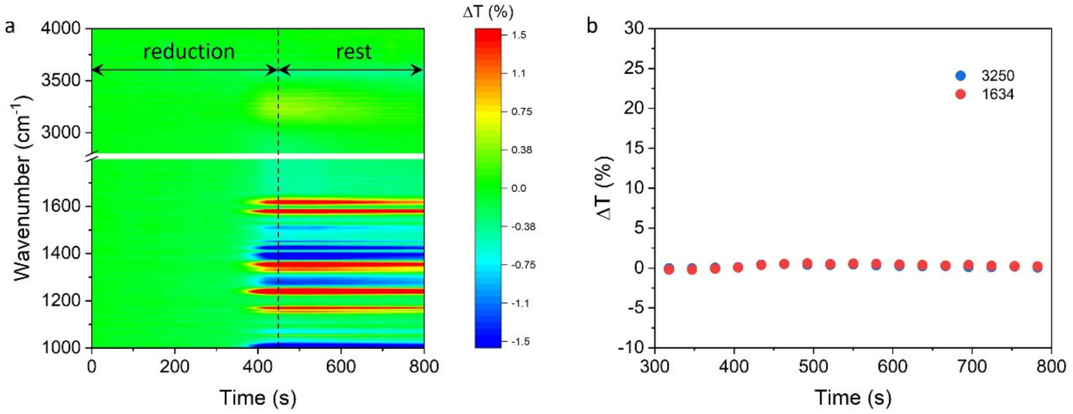

Supplementary Figure 18. a, Evolution of the Operando FTIR spectra of 1,5-DHAQ during reduction and resting processes. b, ΔT vs. time at 1634 cm^{-1} and 3250 cm^{-1}. ΔT is the transmittance difference of the sample relative to that before CV scan. The test was conducted at 25 ±1 °C.

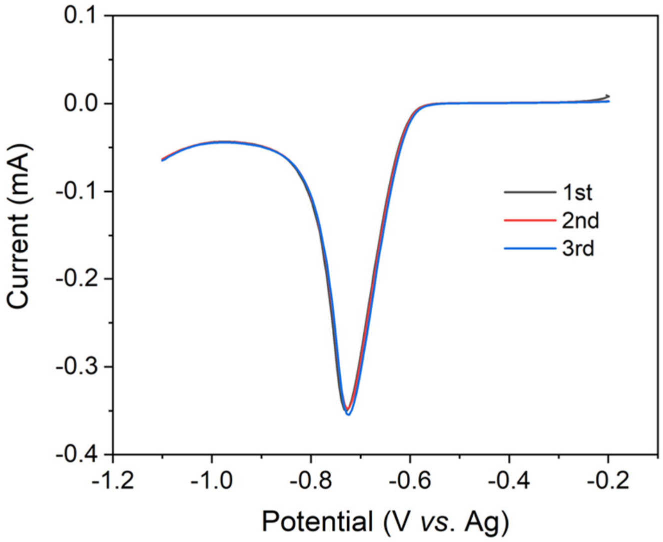

Supplementary Figure 19. Comparison of reduction currents of 1,5-DHAQ during operando FTIR tests. Replot of the reduction current of 1,5-DHAQ during the three consecutive CV scans of FTIR testing. Replot means the currents were overlapped by three reduction process of operando FTIR tests in Supplementary Figures 7 and 17, which were conducted at 25 ±1 °C.

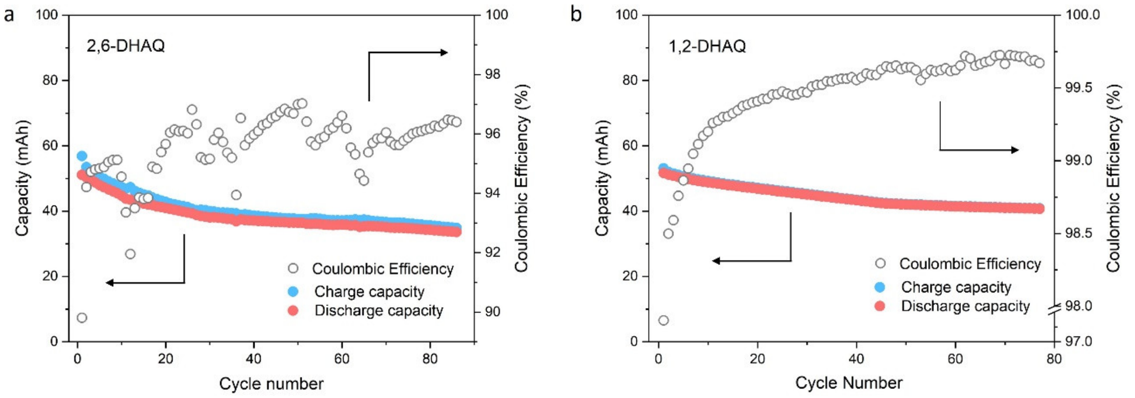

Supplementary Figure 20. Coulombic efficiency and capacity versus cycle number for the anolyte limited DHAQ|$[Fe(CN)_6]^{3-/4-}$ full cells. The catholyte was 80 mL 0.25 M $K_4Fe(CN)_6$ + 0.05 M $K_3Fe(CN)_6$/1M KOH; the anolyte was 10 mL 0.1 M 2,6-DHAQ or 1,2-DHAQ/1M KOH; the current density was 20 mA/cm^2. The total cycling time for 2,6-DHAQ and 1,2-DHAQ based RFBs were 2.83 days and 2.87 days. All tests were conducted at 25 ±1 °C.

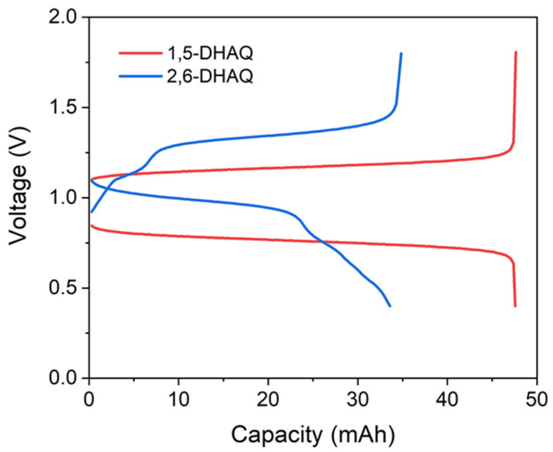

Supplementary Figure 21. Voltage profiles of the last cycle of 1,5-DHAQ and 2,6-DHAQ (86th cycle for 2,6-DHAQ and 73rd cycle for 1,5-DHAQ). The current density was 20 mA/cm^2. All tests were conducted at 25 ±1 °C.

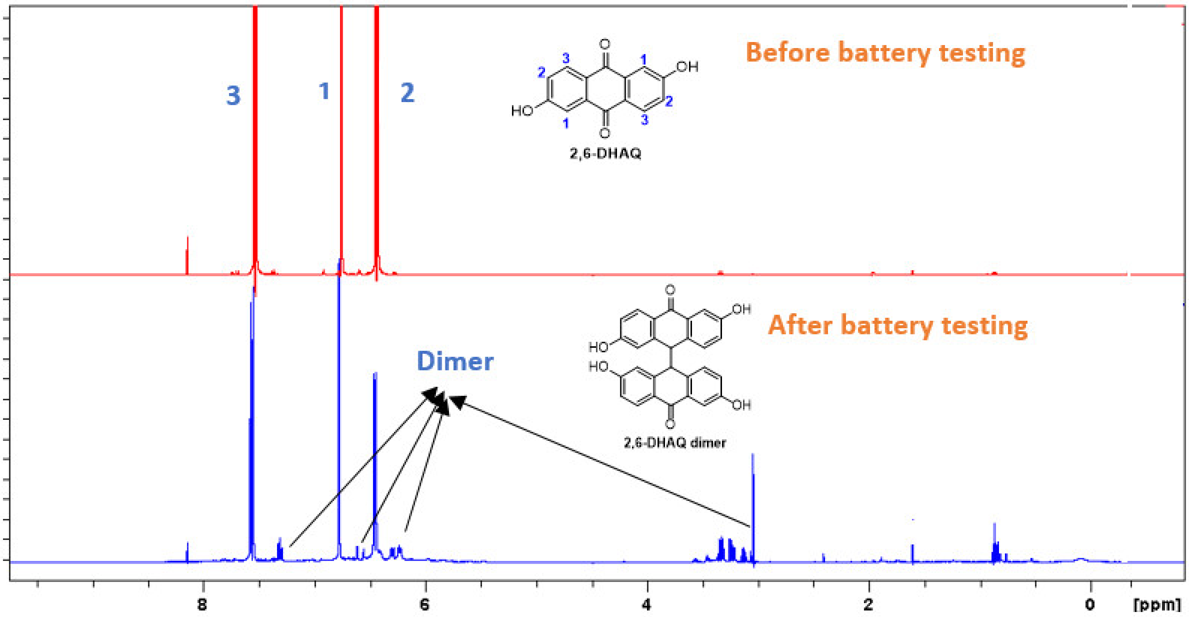

Supplementary Figure 22. Ex situ ^{1}H NMR spectra (500 MHz, D_2O) of 2,6-DHAQ before and after battery testing at the oxidized state. Continued cycling leads to the appearance of additional peaks in the ^{1}H NMR spectrum in D_2O, which belongs to the dimer caused by the reduced 2,6-DHAQ. The structure of 2,6-DHAQ dimer was reported by Goulet et al.[1] The battery was cycled for 86 cycles at the current density of 20 mA/cm^2 and 25 ±1 °C.

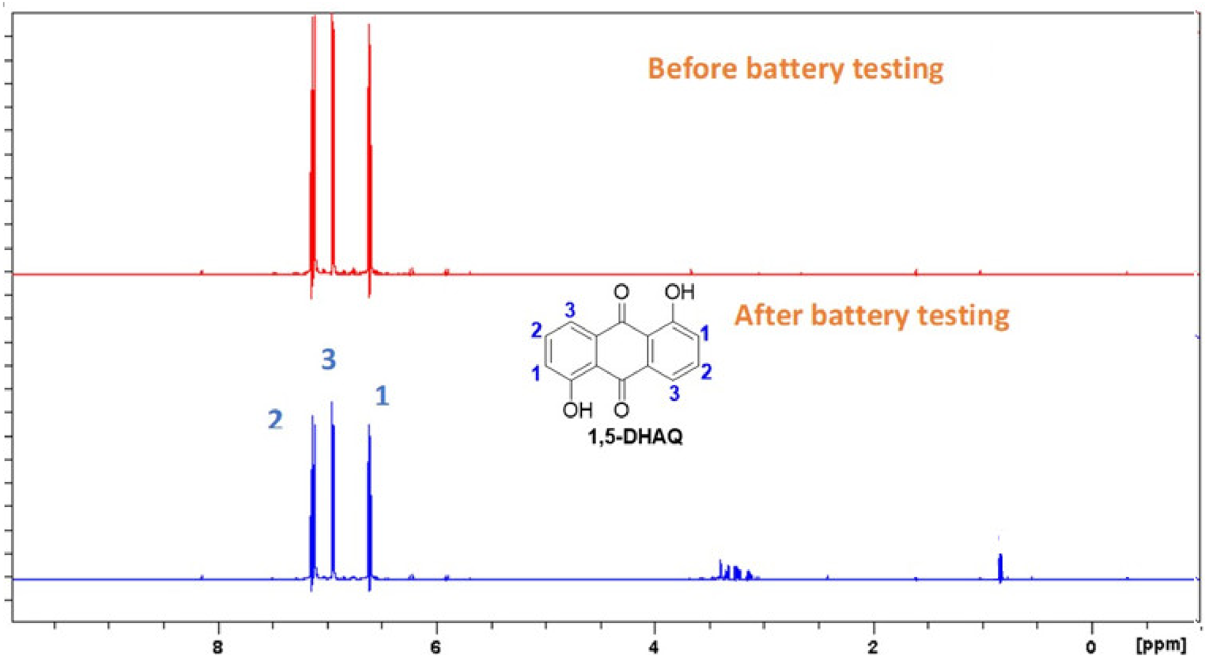

Supplementary Figure 23. Ex situ ^{1}H NMR spectra (500 MHz, D_2O) of 1,5-DHAQ before and after battery testing at the oxidized state. No additional peaks are observed after battery testing, suggesting the highly stability of reduced 1,5-DHAQ. The battery was cycled for 73 cycles at the current density of 20 mA/cm^2 and 25 ±1 °C.

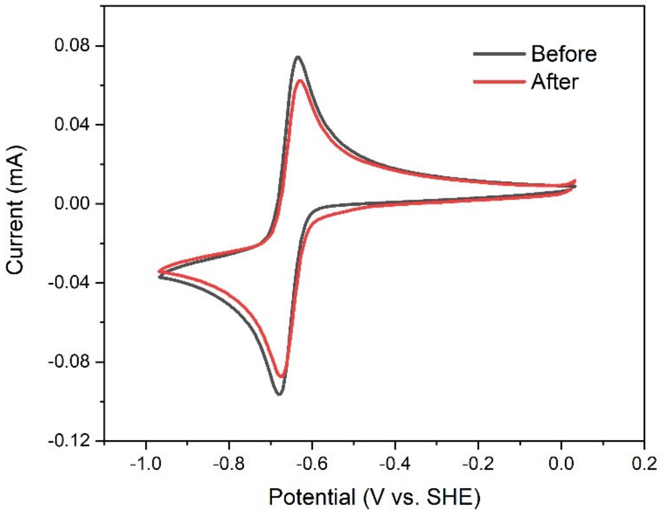

Supplementary Figure 24. CVs of 5 mM diluted 1,2-DHAQ anolyte before and after cycling experiments with a scan rate of 50 mV/s. The 1,2-DHAQ was at the oxidized state for CV tests and the battery was conducted for 77 cycles at the current density of 20 mA/cm^2, after which the anolyte was diluted to conduct the CV test. All the tests were conducted at 25 ±1 °C.

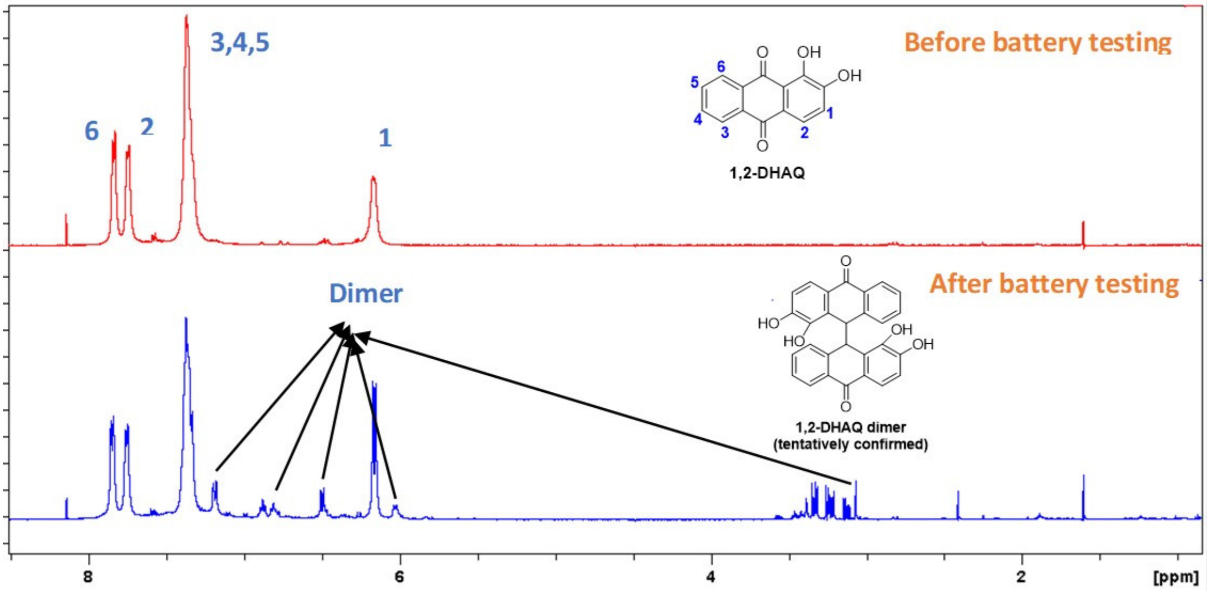

Supplementary Figure 25. Ex situ ^{1}H NMR spectra (500 MHz, D_2O) of 1,2-DHAQ before and after battery testing at the oxidized state. Continued cycling leads to the appearance of additional peaks in the ^{1}H NMR spectrum in D_2O, which belongs to the dimer caused by the reduced 1,2-DHAQ. The structure of 1, 2-DHAQ dimer was tentatively confirmed by presuming it similar to 2,6-DHAQ dimer. The battery was cycled for 77 cycles at the current density of 20 mA/cm^2 and 25 ±1 °C.

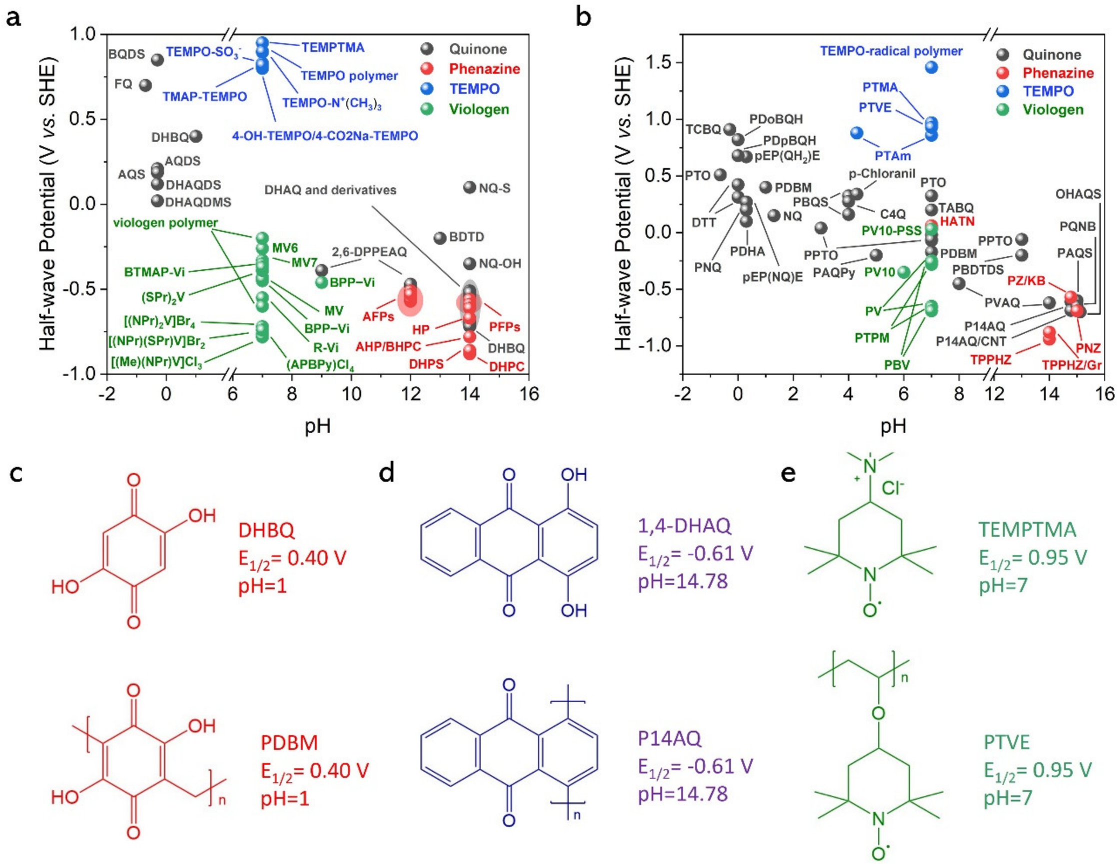

Supplementary Figure 26. Summary of the reported organic materials with the relationship of pH and redox potential. a, Soluble and b, Solid-state redox active species. Their potentials are plotted against pH of the electrolyte solutions. c-e, The soluble redox monomers and their corresponding polymers sharing the same $E_{1/2}$ from acid to alkaline conditions.

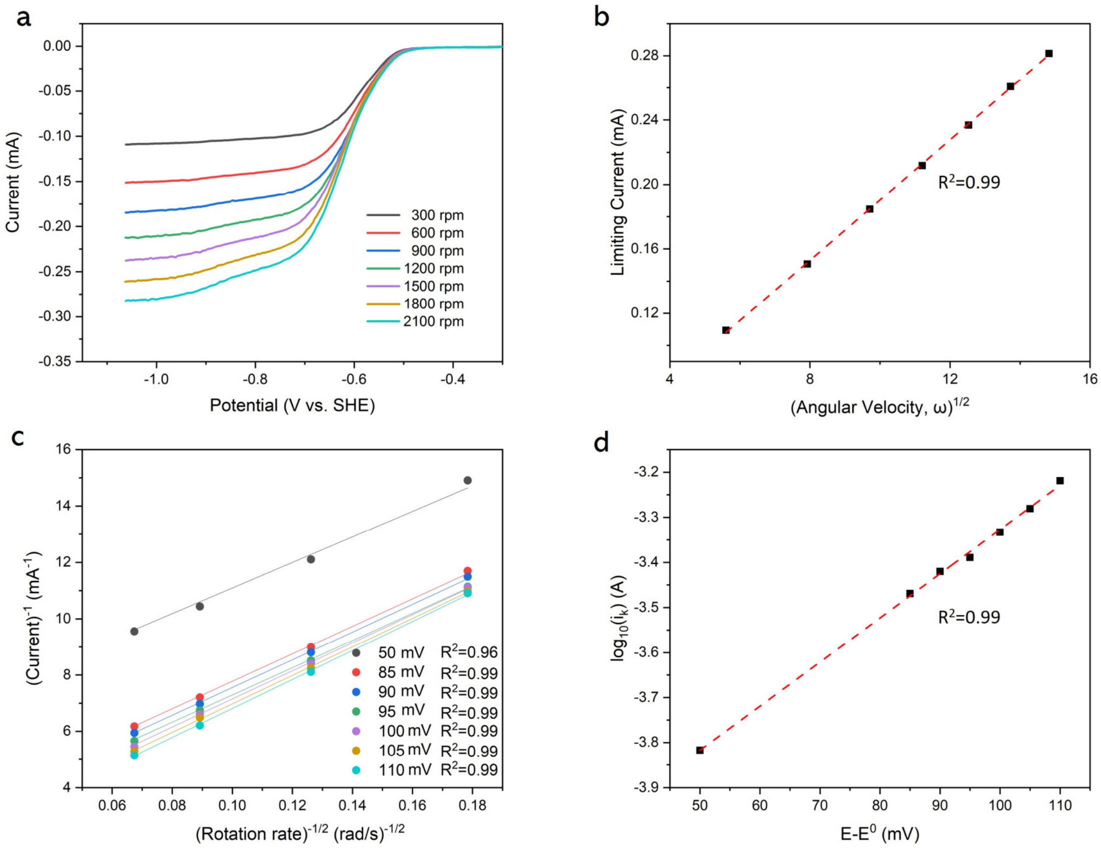

Supplementary Figure 27. 1,5-DHAQ reduction kinetics in rotating disk electrode (RDE). a, Linear sweep voltammograms of 1 mM 1,5-DHAQ in 1 M KOH on a glassy carbon electrode at rotation rates between 300 and 2100 rpm. The small reduction wave might be caused by oxygen. The dissolved oxygen can oxidize the reduced DHAQ which regenerates DHAQ with an EC process, leading to an increase in current (small reduction wave) at around -0.7 V. b, Levich plot (limiting current versus square root of rotation rate in rad/s) of 1 mM 1,5-DHAQ in 1 M KOH. The slope yields a diffusion coefficient for the oxidized form of 1,5-DHAQ of 7.24×10^{-6} cm^2/s. c, Koutecky-Levich plot (reciprocal current versus inverse square root of rotation rate in rad/s) of 1 mM 1,5-DHAQ in 1 M KOH at different overpotentials. d, Fitted Tafel plot of 1 mM 1,5-DHAQ in 1 M KOH. The rate constant is calculated to be 1.29×10^{-3} cm/s. All the tests were conducted at 25 ±1 °C.

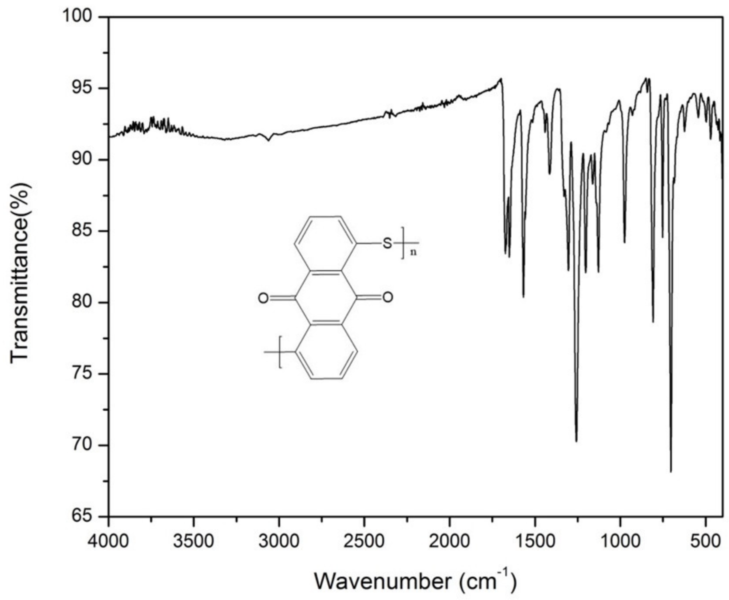

Supplementary Figure 28. FT-IR spectrum of PAQS polymer. The test was conducted at 25 ±1 °C.

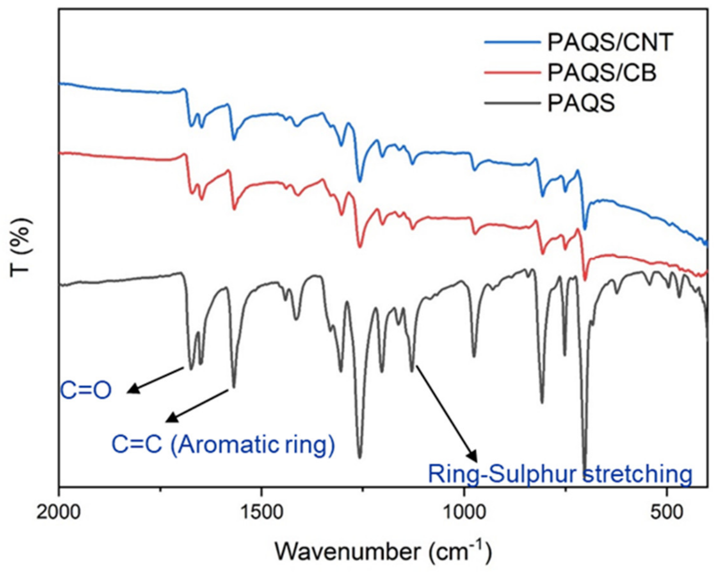

Supplementary Figure 29. FT-IR spectra of PAQS, PAQS/CB and PAQS/CNT. All the tests were conducted at 25 ±1 °C.

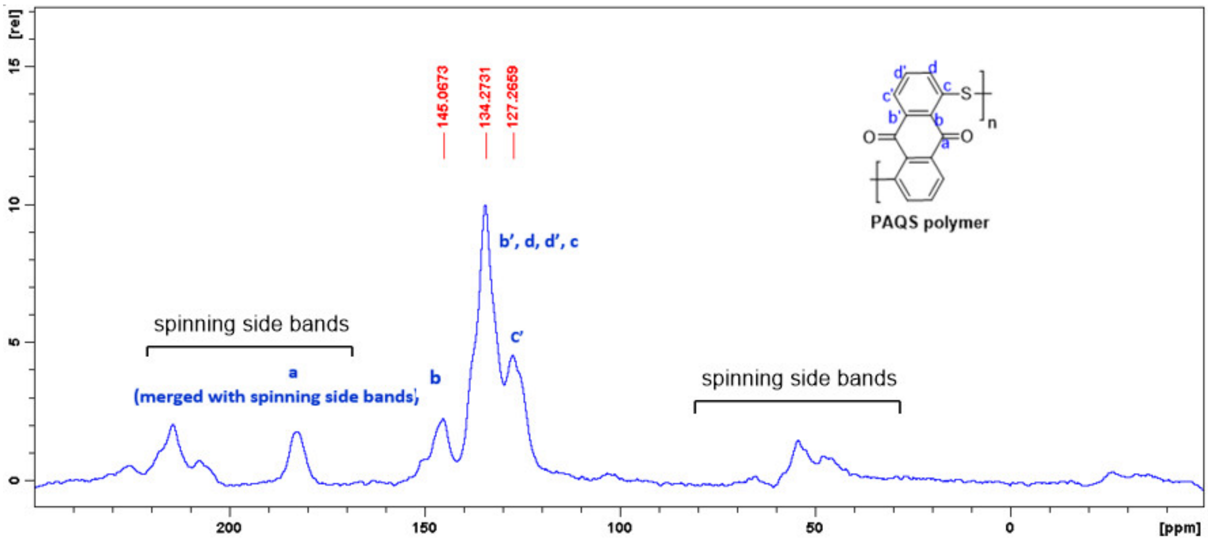

Supplementary Figure 30. Solid-state ^{13}C NMR Spectrum of PAQS polymer.
The PAQS polymer is insoluble in any solvent, thus solid-state ^{13}C NMR spectrum was performed to confirm its structure. Due to the strong rigidity of the polymer chains, the spectral line is significantly widened, and three spinning side bands appear around the main characteristic bands.

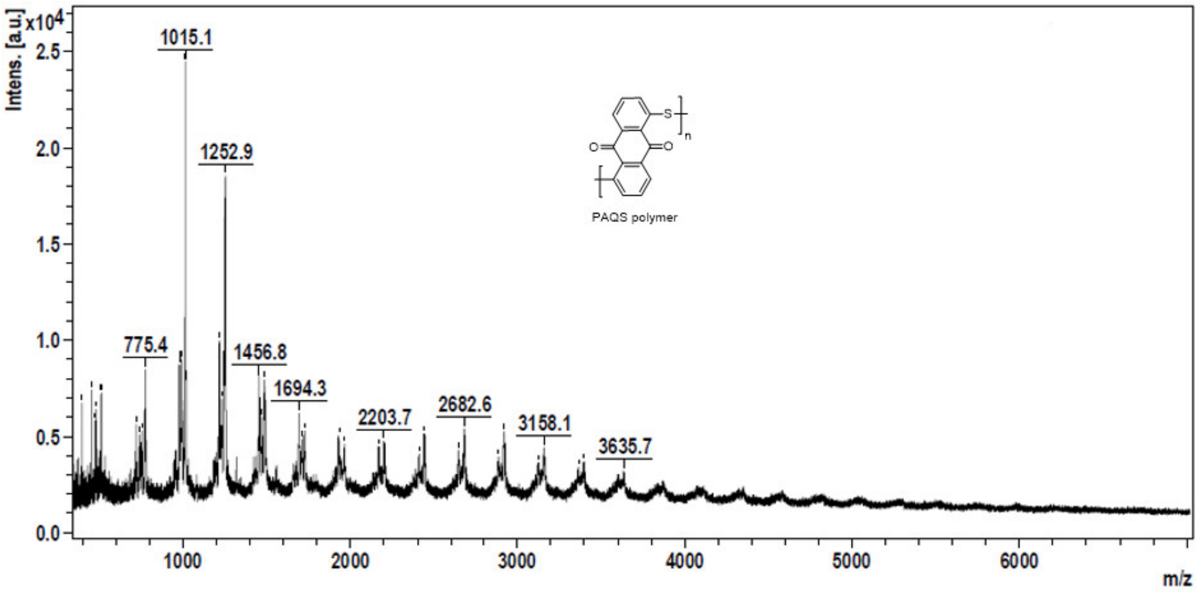

Supplementary Figure 31. Matrix-assisted laser desorption/ionization time-of-flight (MALDI-TOF) mass spectrometry profile of PAQS polymer.

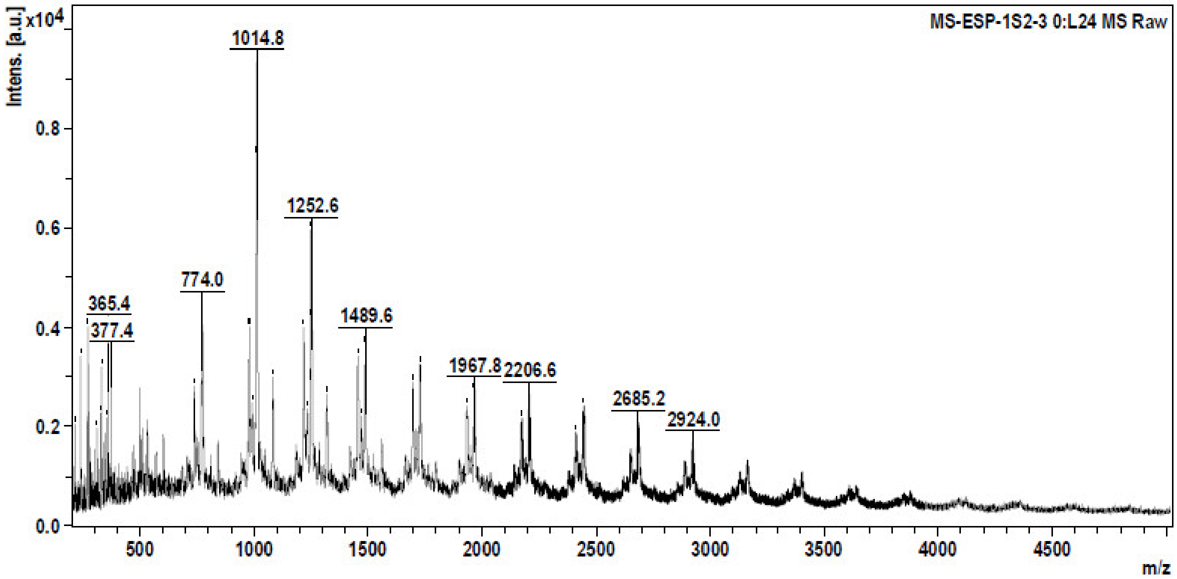

Supplementary Figure 32. Matrix-assisted laser desorption/ionization time-of-flight (MALDI-TOF) mass spectrometry profile of PAQS/CB polymer.

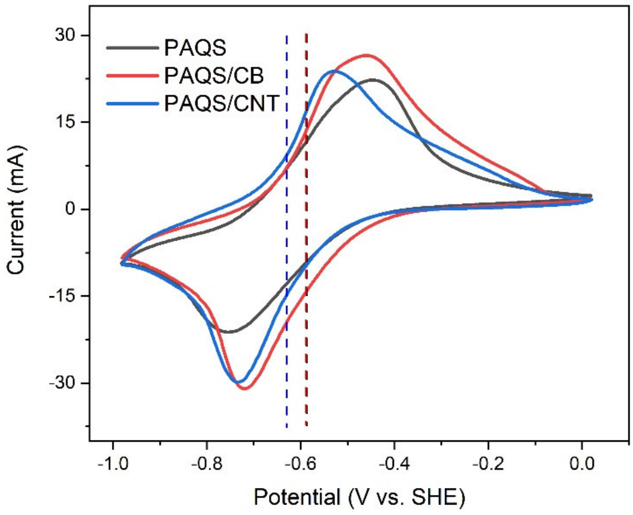

Supplementary Figure 33. CV curves of PAQS, PAQS/CB and PAQS/CNT in 1 M KOH solution at 25 ±1 °C.

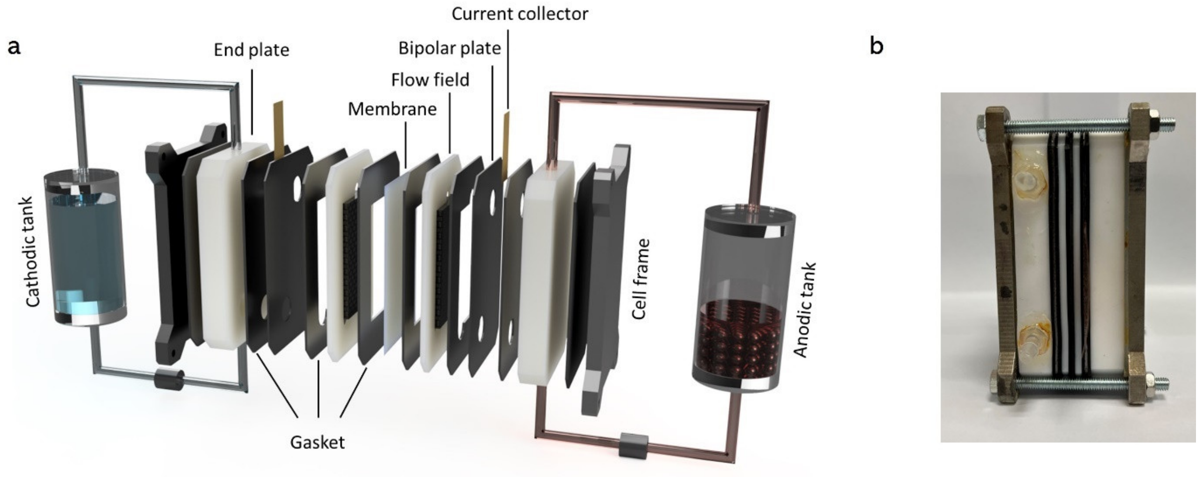

Supplementary Figure 34. a, Schematic of the SMRT-based RFB with electrode active area of 13.5 cm^2. b, The photographic picture of the cell schematic.

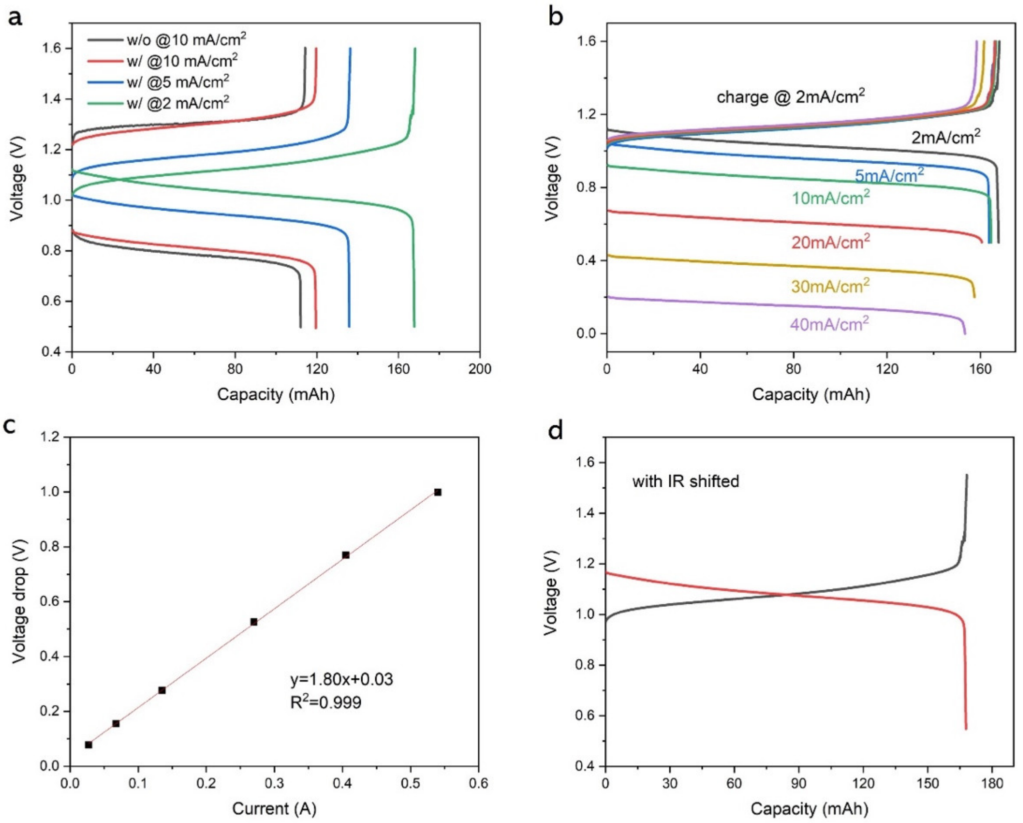

Supplementary Figure 35. Battery performance of SMRT-based 1,5-DHAQ|$[Fe(CN)_6]^{3-/4-}$ full cells. a, Voltage profiles of 0.1 M 1,5-DHAQ|$[Fe(CN)_6]^{3-/4-}$ full cells before (1st cycle) and after adding 312 mAh equivalent capacity of PAQS granules at different charge and discharge current density (2nd to 4th cycle). b, Voltage profiles of the same battery charged at a current density of 2 mA/cm^2 and discharged at different current densities from 2 mA/cm^2 to 40 mA/cm^2 (5th to 10th cycle). c, Plots of voltage drop at different current. d, Replot of the green voltage profile in (a) with IR correction (the values of discharge/charge voltages are modified by plus/minus the value of overpotential caused by the ohmic resistance, respectively. 4th cycle was considered as an example, it is also capable to all other cycles). The catholyte was 80 mL 0.25 M $K_4Fe(CN)_6$ + 0.05 M $K_3Fe(CN)_6$/1M KOH; the anolyte was 20 mL 0.1 M 1,5-DHAQ/1M KOH. The electrode active area was 13.5 cm^2. The liner relationship between voltage and current in (c) indicates that the ohmic resistance from cell stack dominates the voltage loss. All the tests were conducted at 25 ±1 °C.

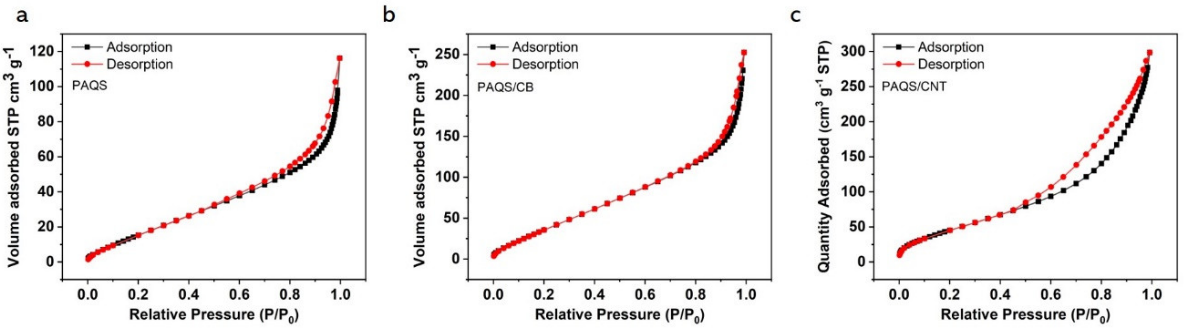

Supplementary Figure 36. N_2-adsorption isotherms of (a) PAQS, (b) PAQS/CB and (c) PAQS/CNT powders.

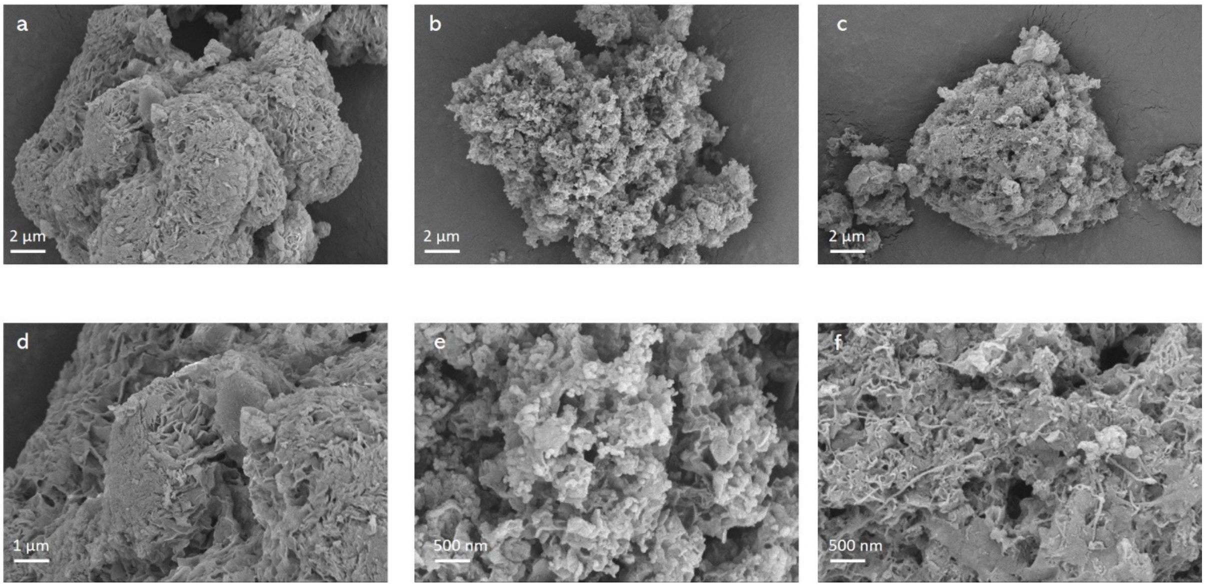

Supplementary Figure 37. SEM images of (a) and (d) PAQS powder, (b) and (e) PAQS/CB composites powder and (c) and (f) PAQS/CNT composites powder. As shown in Supplementary Figures 21e and f, compared with pure PAQS, both CB and CNT are highly dispersed in the PAQS/CB and PAQS/CNT composites, which can increase the electronic conductivity and surface area of the PAQS based materials, promoting the utilization of PAQS.

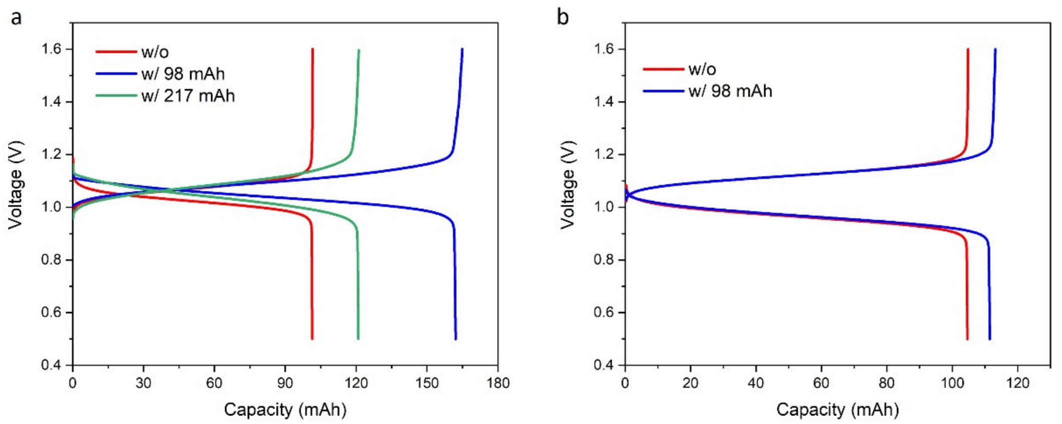

Supplementary Figure 38. a, Voltage profiles of 0.1 M 1,5-DHAQ||$[Fe(CN)_6]^{3-/4-}$ full cells before and after adding 98 or 217 mAh equivalent capacity of PAQS/CB granules at current density of 2 mA/cm^2. b, Voltage profiles of 0.1 M 1,5-DHAQ||$[Fe(CN)_6]^{3-/4-}$ full cells after adding 98 mAh equivalent capacity of PAQS/CNT granules at current density of 2 mA/cm^2. The catholyte was 80 mL 0.25 M $K_4Fe(CN)_6$ + 0.05 M $K_3Fe(CN)_6$/1M KOH; the anolyte was 20 mL 0.1 M 1,5-DHAQ/1M KOH. The electrode active area was 13.5 cm^2. The utilization of PAQS/CB granules increased from 20% to 28% with more granules loading in the tank, the higher utilization could be a result of longer reaction time between 1,5-DHAQ and PAQS/CB solid materials with higher loading in the tank.[2]

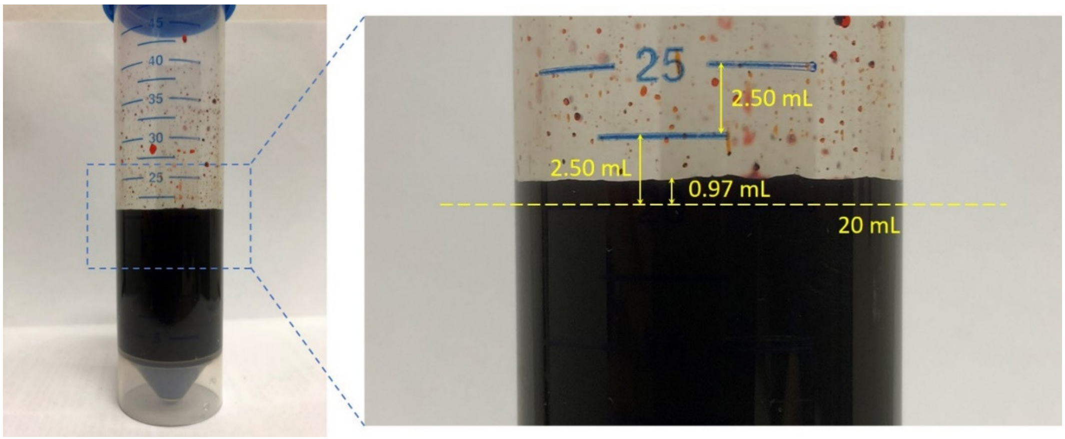

Supplementary Figure 39. Experimental evaluation of theoretical volumetric capacity of PAQS/CB granules. After adding 262.5 mAh equivalent capacity of PAQS/CB granules into 20 mL 0.1 M 1,5-DHAQ/1 M KOH solution, the volume change is 0.97 mL, thus the theoretical volumetric capacity of PAQS/CB granules is calculated to be 271 Ah/L. The details of calculation can be found in Supplementary Note 5.

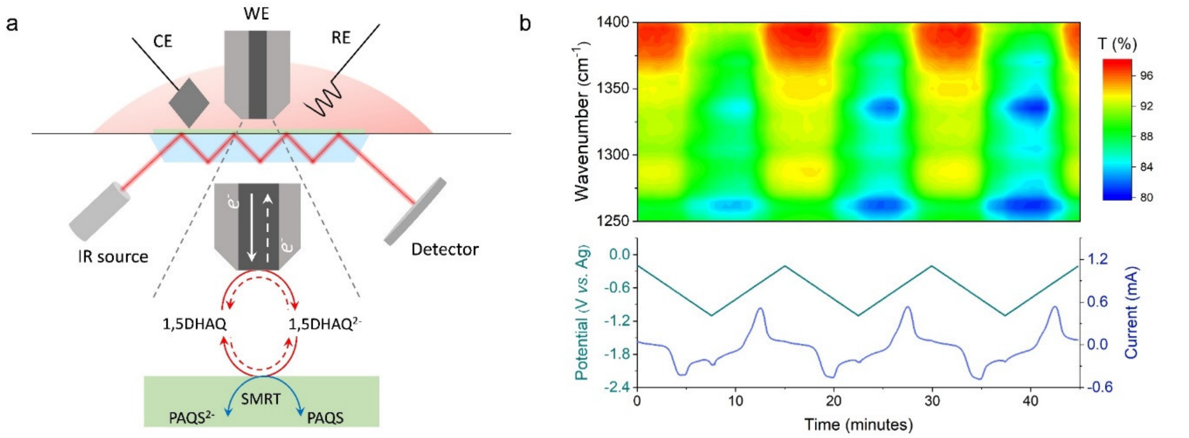

Supplementary Figure 40. (a) Setup for operando ATR-FTIR measurement of PAQS/CB upon SMRT reactions with 1,5-DHAQ. (b) In situ FTIR spectra of PAQS/CB during SMRT reaction with 1,5-DHAQ. Electrolyte: 0.1 M 1,5-DHAQ in 1 M KOH. Background: 1 M KOH. WE: Pt disk; CE: Pt plate; RE: Ag wire. CV scan rate: 2 mV/s. Working distance: 0.05 mm.

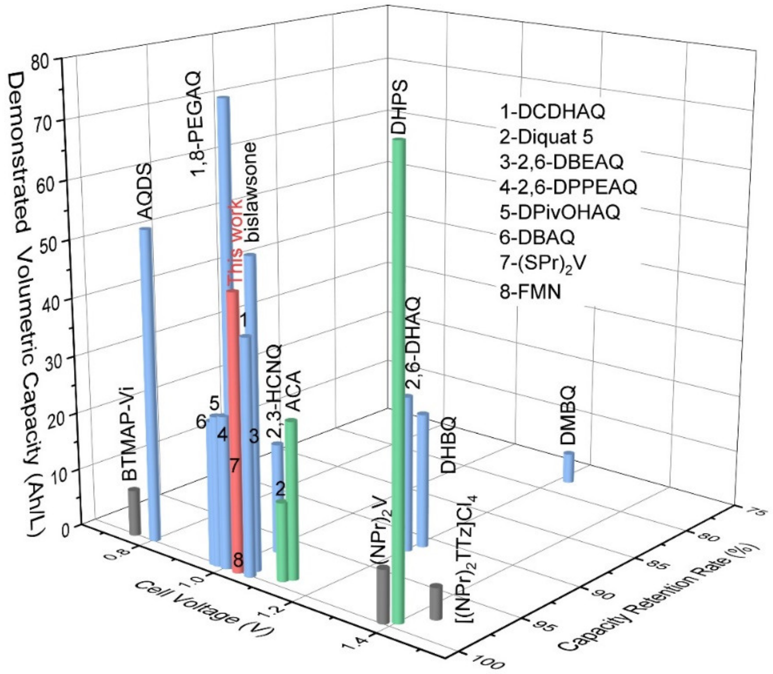

Supplementary Figure 41. Plot of demonstrated volumetric capacity of some reported organic redox mediator-based aqueous anolytes and that of this work, versus capacity retention rate and cell voltage. Blue: quinone-based; green: aza-aromatic reactants-based; grey: viologen-based. The full names of the molecules are listed in the Supplementary Table 4 and detailed information can be found in Supplementary Table 7.

Supplementary Table 1. Summary of the redox potential and solubility of DHAQs in KOH solution.

	Redox potential $E_{1/2}$ (V *vs*. SHE)			Solubility
	1 M KOH	3 M KOH	6 M KOH	1 M KOH
1,2-DHAQ	-0.658	-0.664	-0.666	~0.17 M
1,4-DHAQ	-0.557	-0.585	-0.612	< 10 mM
1,5-DHAQ	-0.561	-0.587	-0.604	~0.15 M
1,8-DHAQ	-0.575	-0.590	-0.595	< 10 mM
2,6-DHAQ	-0.706	-0.684	-0.668	<0.5 M<br><0.7 M (1.5 M KOH)<br><0.8 M (2 M KOH)

Note all the tests were conducted at 25 ±1 °C.

Supplementary Table 2. DFT calculations of the energy for different compounds.

Structure	Energy (Hartree), eV
H_2O	-76.472364
OH^-	-75.978808
2,6-DHAQ^{2-}	-838.771139
2,6-DHAQH$^-$	-764.014912
2,6-DHAQ	-838.572192
2,6-DHAQ$^{2-}\cdot H_2O$	-915.254874
2,6-DHAQ$^{2-}\cdot 2H_2O$	-991.738277
2,6-DHAQ$^{2-}\cdot 3H_2O$	-1068.221321
2,6-DHAQ$^{2-}\cdot 4H_2O$	-1144.704254
2(2,6-DHAQ^{2-})$\cdot H_2O$	-1754.020966
2(2,6-DHAQ^{2-})$\cdot 2H_2O$	-1830.503931
1,5-DHAQ^{2-}	-838.750632
1,5-DHAQH$^-$	-764.007932
1,5-DHAQ	-838.555626
1,5-DHAQ$^{2-}\cdot H_2O$	-915.234979
1,5-DHAQ$^{2-}\cdot 2H_2O$	-991.718845
1,5-DHAQ$^{2-}\cdot 3H_2O$	-1068.200847
1,5-DHAQ$^{2-}\cdot 4H_2O$	-1144.684761
2(1,5-DHAQ^{2-})$\cdot H_2O$	-764.007932
2(1,5-DHAQ^{2-})$\cdot 2H_2O$	-1829.85774
1,2-DHAQ^{2-}	-838.748074
2(1,2-DHAQ^{2-})$\cdot 2H_2O$	-1830.458968
1,4-DHAQ^{2-}	-838.733592
2(1,4-DHAQ^{2-})$\cdot 2H_2O$	-1830.428969
1,8-DHAQ^{2-}	-838.749909
2(1,8-DHAQ^{2-})$\cdot 2H_2O$	-1830.461626

Supplementary Table 3. DFT calculations of the free energy for 1,5-DHAQ based compounds.

Structures	1,5-DHAQ	H_2O	1,5-DHAQH$^-$ (Structure I)	1,5-DHAQH$^-$ (Structure II)	OH^-
Free energy (Hartree), eV	-838.633179	-76.46899	-839.116161	-839.108663	-75.986383

Supplementary Table 4. List of chemicals and their abbreviations

Abbreviation	Full name
P14AQ	poly(1,4-anthraquinone)
PVAQ	poly(2-vinylanthraquinone)
OHAQS	oligohexane anthraquinone sulfide
PTO	pyrene-4,5,9,10-tetraone
PPTO	polymerized version of PTO
PAQS	poly(anthraquinonyl sulfide)
PBDTDS	poly(benzo[1,2-b:4,5-b′]dithiophene-4,8-dione-2,6-diyl sulfide)
PQNB	poly(dianthraquinonesubstitutednorbornene)
TPPHZ	tetrapyridophenazine
PZ/KB	phenazine@Ketjen black composite
PNZ	phenazine
PBQS	poly(benzoquinonyl sulfide)
C4Q	calix[4]quinone
p-Chloranil/TCBQ	tetrachloro-1,4- benzoquinone
HATN	diquinoxalino [2,3-a:2′,3′-c] phenazine
PDBM	poly(2,5-dihydroxy-1,4-benzoquinone-3,6-methylene)
PDBS	poly(2,5-dihydroxy-1,4-benzoquinonylsulfide)
TABQ	tetraamino-p-benzoquinone
PV	polypyrrole-bound viologen
PTPM	polyviologen
PBV	poly(butyl viologen)
PV10-PSS	poly(decyl viologen) and poly(styrene sulfonate)
PTAm	poly(2,2,6,6-tetramethylpiperidinyloxy-4-yl acrylamide)
PTVE	poly(2,2,6,6-tetramethylpiperidinyloxy-4-yl vinylether)
PTMA	poly(2,2,6,6-tetramethylpiperidinyloxy-4-yl)
PDBM	poly(2,5-dihydroxy-1,4-benzoquinone-3,6-methylene)
PDHA	1,8-dihydroxyanthraquinone(DHA)-substituted poly(allylamine)
pEP(NQ)E/ pEP(QH_2)E	p=polymerized; E=3,4-ethylenedioxythiophene; P=3,4-propylenedioxythiophene; NQ=naphthoquinone; NQH_2= naphthohydroquinone
PAQPy	poly(N-anthraquinoyl pyrrole)
DTT	dibenzo[b,i]thian-threne-5,7,12,14-tetraone
NQ	1,4-naphthoquinone
PNQ	poly-(allylamine) yields NQ-substituted poly(allylamine)
PDpBQH	poly(4-(2,5-hydroquinone)-4H-dithieno[3,2-b:2″,3″-d]pyrrole)
PDoBQH	poly(4-(3,4-pyrocatechol)-4H-dithieno[3,2-b:2″,3″-d]pyrrole)
BDTD	benzo[1,2-b:4,5-b′]dithiophene-4,8-dione
2,6-DPPEAQ	(((9,10-dioxo-9,10-dihydroanthracene-2,6-diyl) bis(oxy))bis(propane-3,1-diyl))bis(phosphonic acid)
AMA	alizarin-3-methyliminodiacetic acid
ARS	alizarin red S
2,6-DBEAQ	4,40-((9,10-anthraquinone-2,6-diyl)dioxy)dibutyrate
1,8-DBEAQ	4,40-((9,10-anthraquinone-1,8-diyl)dioxy)dibutyrate
1,2-DBEAQ	4,40-((9,10-anthraquinone-1,2-diyl)dioxy)dibutyrate
DCDHAQ	1,8-dihydroxy-2,7-dicarboxymethyl-9,10-anthraquinone
DPivOHAQ	3,30-(9,10-anthraquinone-diyl)bis(3-methylbutanoic acid)
DBAQ	4,40-(9,10-anthraquinone-diyl)dibutanoic acid
NQ-S	1,2-naphthoquinone-4-sulfonic acid sodium salt
NQ-OH	2-hydroxy-1,4-naphthoquinone

1,6-PFP	3,30-(phenazine-1,6-diyl)dipropionic acid
1,8-PFP	3,30-(phenazine-1,8-diyl)dipropionic acid
2,7-PFP	3,30-(phenazine-2,7-diyl)dipropionic acid
DHPS	7,8-dihydroxyphenazine-2-sulfonic acid
DHPC	7,8-dihydroxyphenazine-2-carboxylic acid
DGAP/DPAP/DAAP /DBAP	amino acid functionalized phenazine
HP	2-hydroxyphenazine
AHP	2-amino-3-hydroxyphenazine
BHPC	benzo[a]hydroxyphenazine-7/8-carboxylic acid
PEGAQ	1,8-bis(2-(2-(2-hydroxyethoxy)ethoxy)-ethoxy)anthracene-9,10-dione
QAAQ	quaternary ammonium protected anthraquinone derivative
TEMPO-$N^+(CH_3)_3$	2,2,6,6-tetramethylpiperidine 1-oxyl derivatives bearing -$N^+(CH_3)_3$ group
TEMPO-SO_3^-	derivative TEMPO-4-sulfate salt
4-OH-TEMPO	4-hydroxy-2,2,6,6-tetramethylpiperidin-1-oxyl
4-CO_2Na-TEMPO	4-carboxylic-2,2,6,6-tetramethylpiperidin-N-oxyl
TEMPTMA	N,N,N-2,2,6,6-heptamethylpiperidinyl oxy-4-ammonium chloride
g^+-TEMPO	cation-grafted TEMPO
TMAP-TEMPO	4-[3-(trimethylammonio)propoxy]-2,2,6,6-tetramethylpiperidine-1-oxyl
(SO_3)V(OH)Br	3-(1′-(2-hydroxyethyl)-[4,4′-bipyridine]-1,1′-diium-1-yl)propane -1-sulfonate bromide
BTMAP-Vi	bis(3-trimethylammonio)propyl viologen tetrachloride
$(SPr)_2V$	1,1′-bis(3-sulfonatopropyl)-4,4′-bipyridinium
MV	methyl viologen
$(APBPy)Cl_4$	1,1′-bis[3-(trimethylamonium)propyl]-4,4′-(1,4 phenylene) bispyridinium tetrachloride
BPP−Vi	1,1′-bis(3-phosphonopropyl)-[4,4′-bipyridine]-1,1′-diium dibromide
MV6	N,N′-dicarbamido-4,4′-bipyridinium dichloride
MV7	N,N′-bis(carboxyethyl)-4,4′-bipyridinium dichloride
$[(NPr)_2TTz]Cl_4$	4,4′-(thiazolo[5,4-d]thiazole-2,5-diyl)bis(1-(3-(trimethylammonio) propyl)pyridin-1-ium) tetrachloride
R-Vi	rod-like sulfonated viologen
$[(Me)(NPr)V]Cl_3$	1-methyl-10-[3-(trimethylammonio)propyl]-4,40-bipyridinium trichloride
$[(NPr)_2V]Br_4$	1,10-bis[3-(trimethylammonio)propyl]-4,40-bipyridinium tetrabromide
$[(NPr)(SPr)V]Br_2$	1-[3-(trimethylammonio)propyl]-10-(3-sulfonatopropyl)-4,40-bipyridinium dibromide
DHBQ	2,5-dihydroxy-1,4-benzoquinone
AQDS	9,10-anthraquinone-2,7-disulphonic acid
AQS	anthraquinone-2-sulfonic acid
DHAQDS	1,8-dihydroxyanthraquinone-2,7-disulfoni cacid
DHAQDMS	1,4-dihydroxyanthraquinone-2,3-dimethylsulfonic acid
FQ	1,4-hydroquinone bearing four (dimethylamino)methyl groups
BQDS	4,5-dihydroxy-1,3-disulfonic acid
DMBQ	2,5-dihydroxy-3,6-dimethyl-1,4-benzoquinone
DHAQ	dihydroxyanthraquinone
2,3-HCNQ	2-hydroxy-3-carboxy-1,4-naphthoquinone
ACA	alloxazine 7/8-carboxylic acid
FMN	flavin mononucleotide
$(NPr)_2V$	1,1′-bis[3-(trimethylammonio)propyl]-4,4′-bipyridinium tetrachloride

Supplementary Table 5. Summary of $E_{1/2}$ of soluble and solid organic redox active species in different pH conditions. The reference numbers in the "Ref" column are associated with the supplementary information file reference list.

molecule	structure	$E_{1/2}$ (V vs. SHE)	pH	Ref.
P14AQ	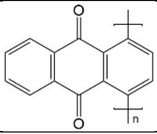	-0.61	14.78	3
P14AQ/CNT		-0.687	14.78	
PVAQ	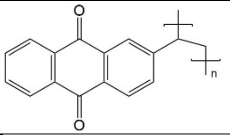	-0.45	8	4
		-0.62	14	
OHAQS	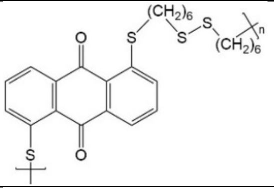 	-0.7	15.11	5
PPTO	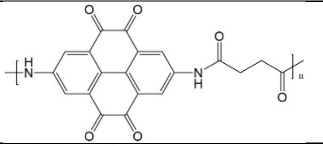	-0.06	13	6
PTO	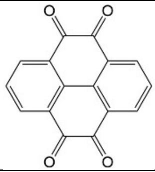	0.325/-0.02	7	
PAQS	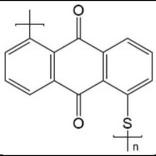	-0.6	15	
PBDTDS	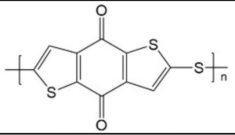	-0.2	13	7
PQNB	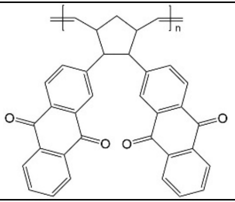	-0.65	15	8
TPPHZ	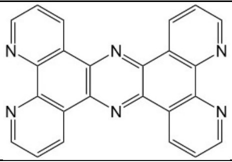	-0.62/-0.94	14	9
TPPHZ/Graphene		-0.88	14	9
PZ/KB	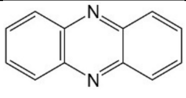	-0.57	14.78	10
PNZ		-0.69	15	11

PBQS	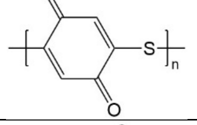	0.325/0.16	Around 4	12
C4Q	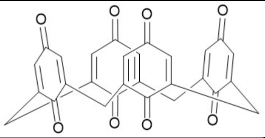	0.275	Around 4	13
p-Chloranil	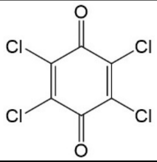	0.34	Around 4.2	14
TCBQ		0.91	-0.3	15
HATN	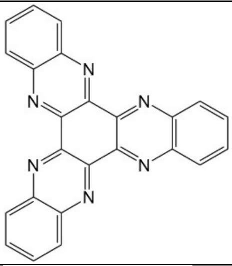	0.06	7	16
PDBM	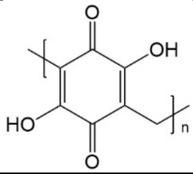	-0.171	7	17
		0.4	1	18
PDBS	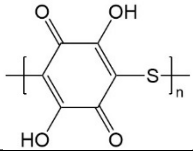	-0.06	7	19
TABQ	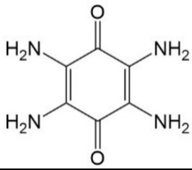	0.04/0.2	7	20
PTPM	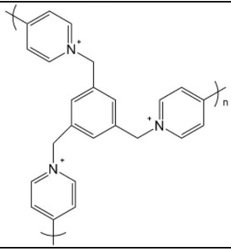	-0.28/-0.66	7	21
PBV	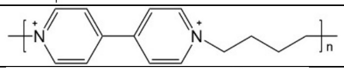	-0.26/-0.69	7	22
PV10	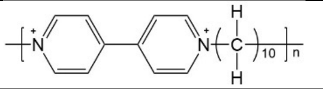	-0.35	Around 7	23
PTAm	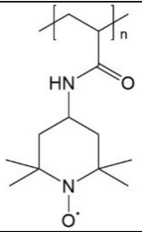	0.86	7	24
		0.88	Around 4.3	23

TEMPO-radical polymer	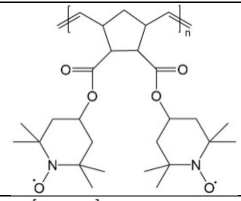	1.46	7	25
PTVE	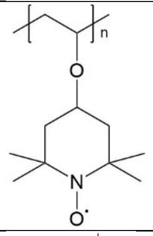	0.95	7	26
PTMA	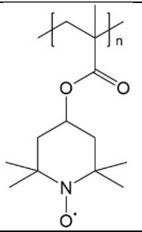	0.97	7	
PDHA	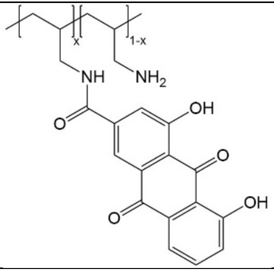	0.1	0.3	27
PDHA/SWNT		0.15	0.3	
PAQPy	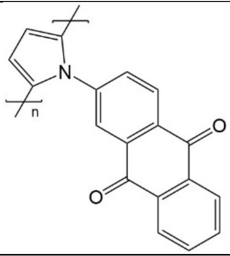	-0.2	5	28
DTT	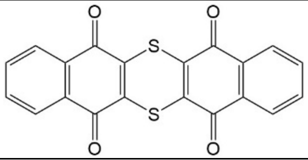	0.424/0.314	0	29
pEP(QH2)E	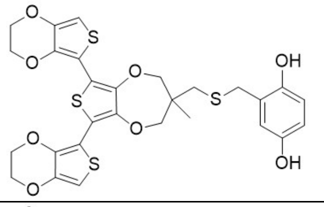	0.67	0.3	30
pEP(NQ)E	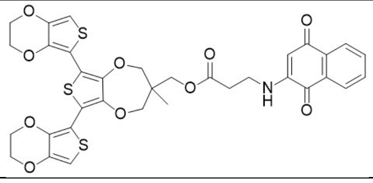	0.27	0.3	

NQ	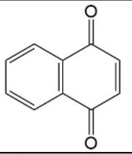	0.15	1.3	31
PNQ	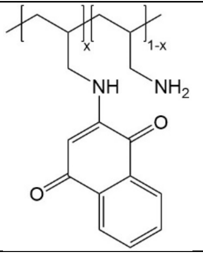	0.2	0.3	
PDpBQH	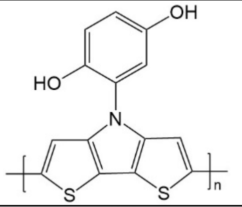	0.68	0	32
PDoBQH	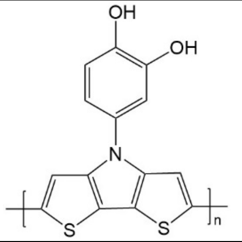	0.82	0	
BTDT	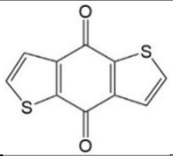	-0.2	13	7
2,6-DPPEAQ	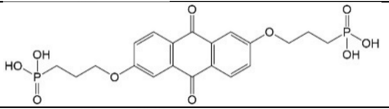	-0.39	9	33
		-0.47	12	
AMA	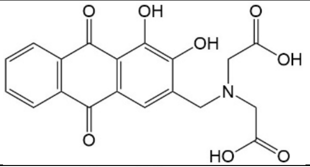	-0.67	14	34
ARS	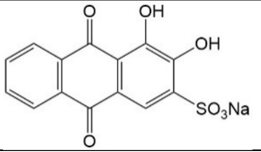	-0.7	14	
2,6-DBEAQ	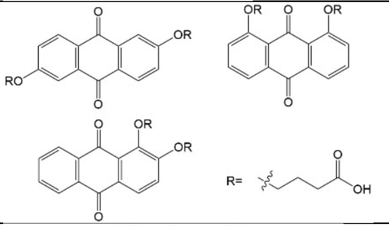	-0.515	14	35
1,8-DBEAQ		-0.51	14	
1,2-DBEAQ		-0.54	14	
DCDHAQ	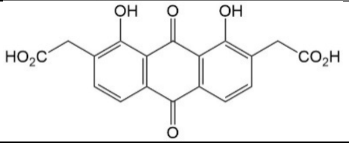	-0.56	14	36

DPivOHAQ	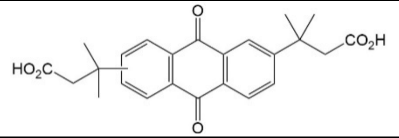	-0.48	12	37
DBAQ	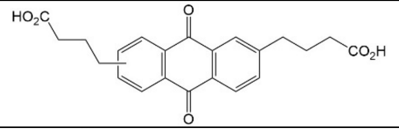	-0.47	12	
DHBQ	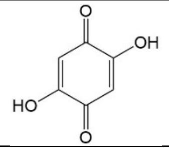	-0.72	14	38
		0.4	1	18
NQ-S	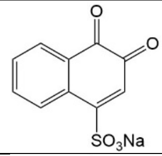	0.1	14	39
NQ-OH	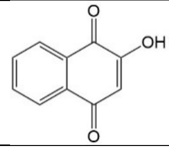	-0.35	14	
1,6-PFP	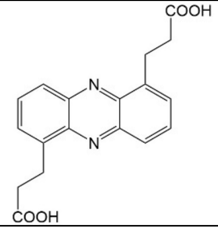	-0.56	14	40
1,8-PFP	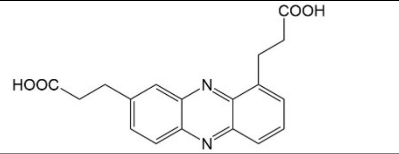	-0.588	14	
2,7-PFP	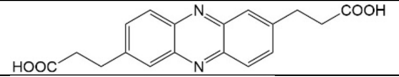	-0.611	14	
DHPC	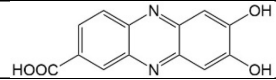	-0.88	14	41
DHPS	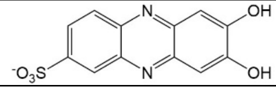	-0.86	14	
2,7-DGAP	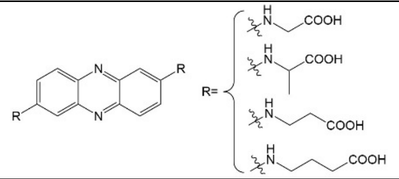	-0.52	12	42
2,7-DAAP		-0.54		
2,7-DPAP		-0.52		
2,7-DBAP		-0.53		
1,8-DGAP	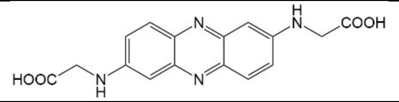	-0.57		
1,6-DGAP	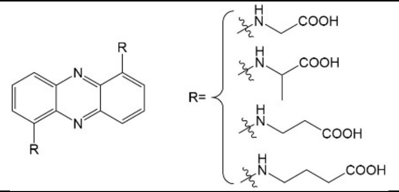	-0.52		
1,6-DAAP		-0.51		
1,6-DPAP		-0.56		
1,6-DBAP		-0.53		

HP	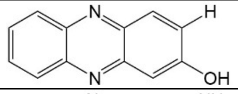	-0.67	14	43
AHP	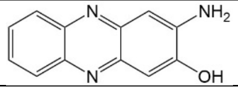	-0.78	14	
BHPC	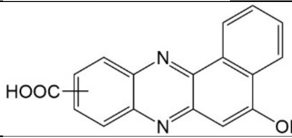	-0.78	14	
PEGAQ	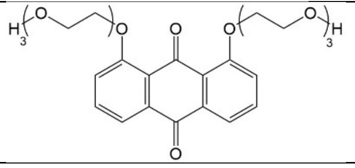	-0.43	7	44
		-0.52	14	
QAAQ	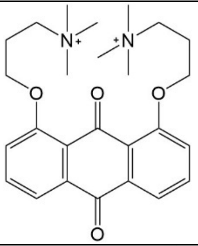	-0.39	7	45
TEMPO-$N^+(CH_3)_3$	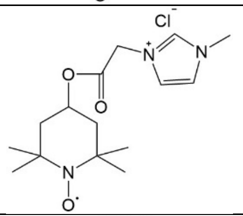	0.895	7	46
TEMPO-SO_3Na	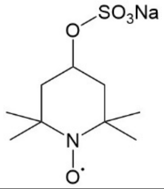	0.83	7	47
4-OH-TEMPO	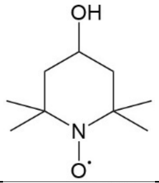	0.8	7	48
4-CO_2Na-TEMPO	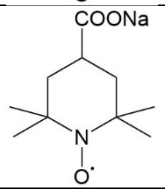	0.8	7	49
TEMPTMA	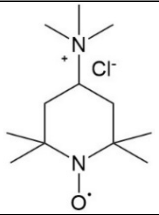	0.95	7	50

g⁺-TEMPO	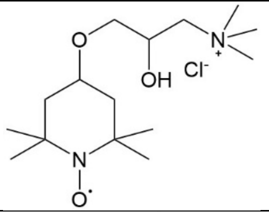	0.82	7	51
TEMPO-containing polymer	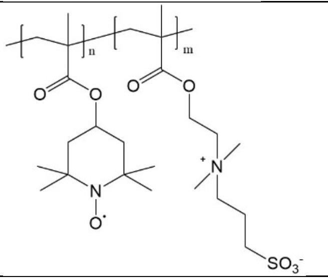	0.9	7	52
	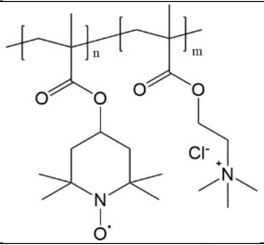	0.9	7	53
TMAP-TEMPO	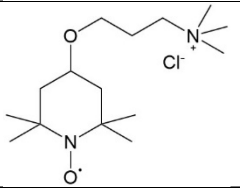	0.81	7	54
$(SO_3)V(OH)Br$	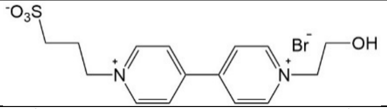	-0.78/-0.39	7	47
BTMAP-Vi	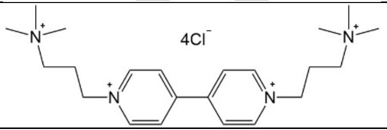	-0.358 V	7	55
$(SPr)_2V$	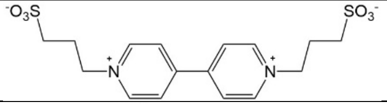	-0.43	7	56
MV	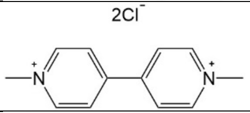	-0.45	7	48
$(APBPy)Cl_4$	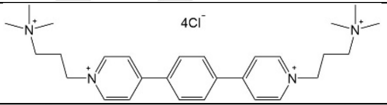	-0.763	7	57
BPP−Vi	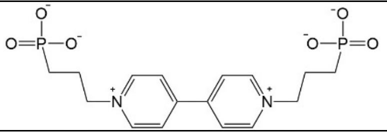	-0.46	9	58
MV6	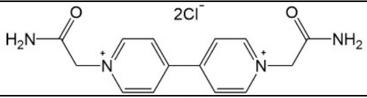	-0.26	7	50
MV7	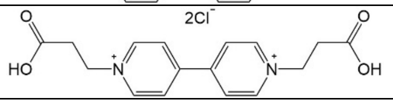	-0.33	7	

$[(NPr)_2TTz]Cl_4$	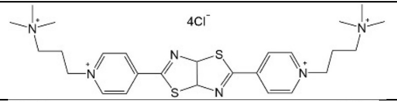	-0.44	7	59
R-Vi	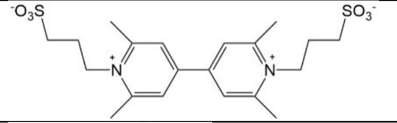	-0.55	7	60
soluble viologen polymer	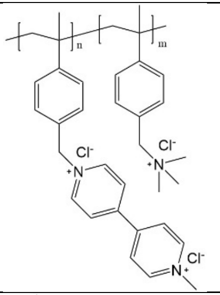	-0.6/-0.2	7	53
$[(Me)(NPr)V]Cl_3$	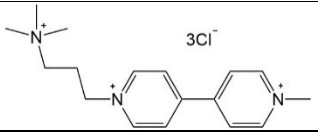	-0.78/-0.39	7	61
$[(NPr)_2V]Br_4$	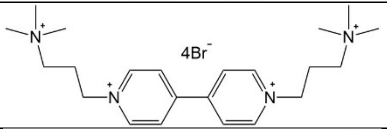	-0.72/-0.35	7	
$[(NPr)(SPr)V]Br_2$	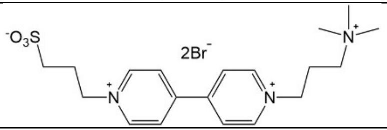	-0.74/-0.37	7	
AQDS	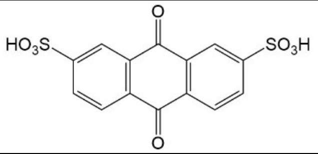	0.21	-0.3	62
AQS	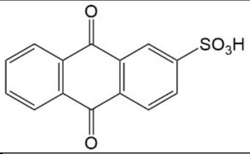	0.187	-0.3	63
DHAQDS	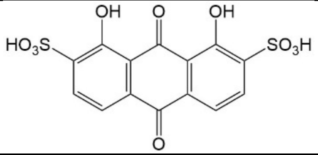	0.12	-0.3	
DHAQDMS	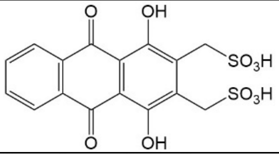	0.02	-0.3	
FQ	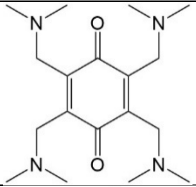	0.7	-0.7	64
BQDS	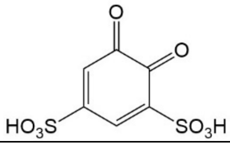	0.85	-0.3	65

Supplementary Table 6. Comparison of 1,5-DHAQ/PAQS/CB anolyte system, DHBQ and DPivOHAQ anolytes. The tests of 1,5-DHAQ/PAQS/CB anolyte were conducted at 25 ±1 °C.

	1,5-DHAQ/PAQS/CB	DHBQ	DPivOHAQ	Parameters grade (1-6)
Stability (capacity fade rate)	0.02%/day	9%/day	0.0018%/day	6: ≤0.01%/day<br>5: 0.01%-0.02%/day<br>1:>5%/day
Voltage (paired with $[Fe(CN)_6]^{3-/4-}$	1.03 V	1.21 V	0.98 V	6:>1.1 V<br>5:>1.0 V<br>4:>0.9 V
Demonstrated Volumetric capacity	44.6 Ah/L	23.2 Ah/L	25.6 Ah/L	6:>150 Ah/L; 5:100-150 Ah/L; 4:60-100 Ah/L; 3:30-60 Ah/L; 2:10-30 Ah/L; 1:<10 Ah/L
Availability	virtually infinite	virtually infinite	virtually infinite	
Duration	>48 days	Around 4 days	15.6 days	6:>40 days; 5>30 days<br>4:>20 days; 3:>10 days
Kinetics (rate constant)	1.29×10^{-3} cm/s	2.12×10^{-3} cm/s[38]	2.48×10^{-3} cm/s[37]	6:> 3×10^{-3} cm/s<br>5: 2×10^{-3} - 3×10^{-3} cm/s<br>4: 1×10^{-3} - 2×10^{-3} cm/s

Supplementary Table 7. Summary of results from full cells containing organic-based anolytes.

Name	Capacity fade rate (%/day)	Demonstrated/Theoretical volumetric capacity (Ah/L)	Testing time (days)	Redox potential (V vs. SHE)/Cell voltage (V)	References
DMBQ	22	5.36/N.A.	Around 3	-0.75/1.22	66
DHBQ	9	23.15/231	Around 4	-0.72/1.21	38
2,6-DHAQ	8	26.8/32.2	Around 1.79	-0.68/1.20	67
bislawsone	0.74	52.68/60	19.6	-0.55/1.05	68
2,3-HCNQ	3.4	18.8/64.3	Around 2.23	-0.53/1.02	69
1,8-PEGAQ	0.5	76.94/120	18	-0.43/1.00	44
2,6-DBEAQ	0.05	22.89/50.6	5	-0.54/1.06	35
2,6-DPPEAQ	0.014	26/40.2	12.3	-0.47/1.00	33
DHPS	0.68	75.04/96.5	Around 15.63	-0.81/1.40	41
ACA	1.2	26.8/108	Around 5.36	-0.62/1.13	70
FMN	0.6	0.13/80.4	5.17	-0.5/1.03	71
DPivOHAQ	0.0018	25.6/37.5	15.6	-0.48/0.98	37
DBAQ	0.0084	24.58/53.6	15.5	-0.47/0.97	37
DCDHAQ	0.03	40.2/70	9	-0.56/1.06	36
AQDS	0.19	53.6/>53.6	Around 4.17	0.21/0.80	62
Diquat 5	0.73	13.4/72.4	24.88	-0.51/1.12	72
$[(NPr)_2TTz]Cl_4$	2.25	5.36/60	Around 4.02	-0.44/1.44	59
$(SPr)_2V$	0.88	13.4/53.6	Around 6.7	-0.43/1.00	56
BTMAP-Vi	0.033	8/53.6	16.6	-0.36/0.75	55
$(NPr)_2V$	0.2	9/N.A.	Around 11.17	-0.38/1.38	73
1,5-DHAQ-PAQS/CB system	0.02	47/139.5	More than 48	-0.56/1.03	This work

The testing time is calculated without considering the capacity fade and molecule utilization during the whole cycling process, so the actual time is less then calculated value, here we used "around" to indicate the results. Without the indicator "around", then the testing time is indicated in the reference paper. And the demonstrated volumetric capacity is based on the first cycle without considering the capacity fade. For 1,5-DHAQ-PAQS/CB system, the tests were conducted at 25 ±1 °C.

Supplementary Note 1. The adsorption energy calculation of different complex.

The energy of different compounds was summarized in Supplementary Table 2. The adsorption energy of one H_2O on one 2,6-$DHAQ^{2-}$ is calculated by $E_{ad} = \left(E_{2,6-\mathrm{DHAQ}^{2-}\cdot \mathrm{nH_2O}} - E_{2,6-\mathrm{DHAQ}^{2-}} - nE_{\mathrm{H_2O}}\right)/n$, which are -0.310, -0.305, -0.300 and -0.297 eV for 2,6-$DHAQ^{2-}\cdot H_2O$, 2,6-$DHAQ^{2-}\cdot 2H_2O$, 2,6-$DHAQ^{2-}\cdot 3H_2O$ and 2,6-$DHAQ^{2-}\cdot 4H_2O$, respectively. The negative adsorption energy means that H_2O can spontaneously adsorb onto 2,6-$DHAQ^{2-}$. Considering the adsorption energy of one water molecule onto one 2,6-$DHAQ^{2-}$ is the largest, here we use 2,6-$DHAQ^{2-}\cdot H_2O$ for the further analysis.

The adsorption energy of one H_2O on 2,6-$DHAQ^{2-}\cdot H_2O$ is $E_{ad} = \left(E_{2,6-\mathrm{DHAQ}^{2-}\cdot 2\mathrm{H_2O}} - E_{2,6-\mathrm{DHAQ}^{2-}\cdot H_2O} - E_{\mathrm{H_2O}}\right) \times 27.2 = -0.3\, eV$ and one 2,6-$DHAQ^{2-}$ on 2,6-$DHAQ^{2-}\cdot H_2O$ is $E_{ad} = \left(E_{2(2,6-\mathrm{DHAQ}^{2-})\cdot \mathrm{H_2O}} - E_{2,6-\mathrm{DHAQ}^{2-}} - E_{2,6-DHAQ^{2-}\cdot H_2O}\right) \times 27.2 = 0.14\, eV$, indicating a preference of adsorption another H_2O molecule. The adsorption energy of one 2,6-$DHAQ^{2-}$ on 2,6-$DHAQ^{2-}\cdot 2H_2O$ is $E_{ad} = \left(E_{2(2,6-\mathrm{DHAQ}^{2-})\cdot 2\mathrm{H_2O}} - E_{2,6-\mathrm{DHAQ}^{2-}} - E_{2,6-\mathrm{DHAQ}^{2-}\cdot 2\mathrm{H_2O}}\right) \times 27.2 = 0.15\, eV$, indicating there is an energy barrier of adsorption another 2,6-$DHAQ^{2-}$ for both routes.

The energy change of disproportionation reaction of 2,6-$DHAQ^{2-}$ is $\Delta E_{dis} = \left(E_{2,6-DHAQ} + E_{2,6-\mathrm{DHAQH}^-} + 3 \times E_{OH^-} - E_{2(2,6-\mathrm{DHAQ}^{2-})\cdot 2\mathrm{H_2O}}\right) \times 27.2 = -0.53\, eV$, indicating the spontaneous transformation from 2(2,6-$DHAQ^{2-}$)$\cdot$$2H_2O$ to 2,6-$DHAQ^{2-}$ and 2,6-DHA. Based on this analysis, the disproportionation reaction can spontaneously occur once the 2(2,6-$DHAQ^{2-}$)$\cdot$$2H_2O$ formed, and adsorption of the second 2,6-$DHAQ^{2-}$ molecule is the rate determining step for the whole reaction process.

The adsorption energy of one H_2O on one 1,5-$DHAQ^{2-}$ is $E_{ad} = \left(E_{1,5-\mathrm{DHAQ}^{2-}\cdot \mathrm{H_2O}} - E_{1,5-\mathrm{DHAQ}^{2-}} - E_{\mathrm{H_2O}}\right)$, which are -0.326, -0.319, -0.300 and -0.303 eV for 1,5-$DHAQ^{2-}\cdot H_2O$, 1,5-$DHAQ^{2-}\cdot 2H_2O$, 1,5-$DHAQ^{2-}\cdot 3H_2O$ and 1,5-$DHAQ^{2-}\cdot 4H_2O$, respectively. The negative adsorption energy means that H_2O can spontaneously adsorb onto 1,5-$DHAQ^{2-}$. Considering the adsorption energy of one water molecule onto one 1,5-$DHAQ^{2-}$ is the largest, here we use 1,5-$DHAQ^{2-}\cdot H_2O$ for the further analysis.

The adsorption energy of one H_2O onto 1,5-$DHAQ^{2-}\cdot H_2O$ is $E_{ad} = \left(E_{1,5-\mathrm{DHAQ}^{2-}\cdot 2\mathrm{H_2O})} - E_{1,5-\mathrm{DHAQ}^{2-}\cdot \mathrm{H_2O}} - E_{\mathrm{H_2O}}\right) \times 27.2 = -0.31\, eV$. And one 1,5-$DHAQ^{2-}$ on 1,5-$DHAQ^{2-}\cdot H_2O$ is $E_{ad} = \left(E_{2(1,5-\mathrm{DHAQ}^{2-})\cdot \mathrm{H_2O}} - E_{1,5-\mathrm{DHAQ}^{2-}\cdot \mathrm{H_2O}} - E_{\mathrm{H_2O}}\right) \times 27.2 = 15.92\, eV$, indicating a preference of adsorption another H_2O molecule. The adsorption energy of one 1,5-$DHAQ^{2-}$ on 1,5-$DHAQ^{2-}\cdot 2H_2O$ is $E_{ad} = \left(E_{2(1,5-\mathrm{DHAQ}^{2-})\cdot 2\mathrm{H_2O}} - E_{2,6-\mathrm{DHAQ}^{2-}} - E_{2,6-\mathrm{DHAQ}^{2-}\cdot 2\mathrm{H_2O}}\right) \times 27.2 = 16.64\, eV$. Such huge energy barrier of adsorption another 1,5-$DHAQ^{2-}$ indicated that 1,5-$DHAQ^{2-}\cdot H_2O$ is the only stable state, thus preventing the disproportionation reaction.

The adsorption energy of two H_2O on two 1,2-$DHAQ^{2-}$ is $E_{ad} = \left(E_{2(1,2-\mathrm{DHAQ}^{2-}\cdot \mathrm{H_2O})} - 2 \times E_{1,2-\mathrm{DHAQ}^{2-}} - 2 \times E_{\mathrm{H_2O}}\right) \times 27.2 = -0.49\, eV$. The negative adsorption energy means that H_2O can spontaneously adsorb onto 1,2-$DHAQ^{2-}$.

The adsorption energy of two H_2O on two 1,4-$DHAQ^{2-}$ is $E_{ad} = \left(E_{2(1,4-\mathrm{DHAQ}^{2-}\cdot \mathrm{H_2O})} - 2 \times E_{1,4-\mathrm{DHAQ}^{2-}} - 2 \times E_{\mathrm{H_2O}}\right) \times 27.2 = -0.46\, eV$. The negative adsorption energy means that H_2O can spontaneously adsorb onto 1,4-$DHAQ^{2-}$.

The adsorption energy of two H_2O on two 1,8-$DHAQ^{2-}$ is $E_{ad} = \left(E_{2(1,8-\mathrm{DHAQ}^{2-}\cdot \mathrm{H_2O})} - 2 \times E_{1,8-\mathrm{DHAQ}^{2-}} - 2 \times E_{\mathrm{H_2O}}\right) \times 27.2 = -0.46\, eV$. The negative adsorption energy means that H_2O can spontaneously adsorb onto 1,8-$DHAQ^{2-}$.

Supplementary Note 2. Protonation analysis of reduced 1,5-DHAQ.

The possibility of protonation of reduced 1,5-DHAQ was analyzed by DFT calculations (Supplementary Table 3). Considering the symmetric structure of reduced 1,5-DHAQ, the protonation process may take place at two positions as shown in Supplementary Figure 15. The energy of Structure I is lower than Structure II, suggesting Structure I is more stable and is thus used for the following analysis. The free energy change of the protonation process of Structure I was calculated based on the following equation in 1 M KOH solution:

$$1{,}5\text{-}DHAQ^{2-} + H_2O \rightarrow 1{,}5\text{-}DHAQH^{-} + OH^{-}$$

The pH of the electrolyte would vary with the different protonation process of 1,5-DHAQ. If there isn't protonation, then the protons from 0.1 M 1,5-DHAQ will all react with OH^- in the electrolyte, resulting in a pH of 13.9 (without considering the volume change); if the 1,5-DHAQ is totally protonated, then there will be no proton dissolved into the solution and the pH of 1 M KOH remains 14. Thus, the pH could be in the range of 13.9-14 after dissolving 0.1 M 1,5-DHAQ in 1 M KOH. Then the ΔG was calculated with pH corrections:[74]

$$\Delta G = G_{1,5\text{-}DHAQH^-} + G_{OH^-} - G_{1,5\text{-}DHAQ^{2-}} - H_2O + pH \times k_B T \ln 10$$

where k_B is the Boltzmann constant and T is the temperature. This leads to a ΔG>0.81 eV, indicating the protonation is not energetically favorable. Instead, the reduced 1,5-DHAQ would become deprotonated at such a high pH.

The above seemingly results in a pH-independent redox potential of 1,5-DHAQ, while the CV measurement in Figure S3 indicates a negative shift of the redox potential at high pH. This could be rationalized by the changes of activity coefficient. Based on the Nernst equation:

$$E = E^0 - \frac{RT}{nF}\ln\left(\frac{\alpha_{red}}{\alpha_{ox}}\right) = E^0 - \frac{RT}{nF}\ln\left(\frac{\gamma_{red}}{\gamma_{ox}}\right) - \frac{RT}{nF}\ln\left(\frac{C_{red}}{C_{ox}}\right) = E_f^0 - \frac{RT}{nF}\ln\left(\frac{C_{red}}{C_{ox}}\right)$$

Where E^0 is the standard potential, α is the activity of the reduced and oxidized species, γ is the activity coefficient of the reduced and oxidized species, C is the concentration of reduced and oxidized species, and E_f^0 is the formal potential:

$$E_f^0 = E^0 - \frac{RT}{nF}\ln\left(\frac{\gamma_{red}}{\gamma_{ox}}\right)$$

Considering that:

$$E_{1/2} = E_f^0 + \frac{RT}{nF}\ln\left(\frac{D_{red}}{D_{ox}}\right)^{0.5}$$

Assuming $D_{red} = D_{ox}$, then:

$$E_{1/2} = E_f^0 = E^0 - \frac{RT}{nF}\ln\left(\frac{\gamma_{red}}{\gamma_{ox}}\right)$$

As a result, $E_{1/2}$ may vary with the activity coefficients of the reduced and oxidized species. For different pH conditions: 1 M KOH, 3 M KOH and 6 M KOH, the changes of supporting electrolyte concentration or ionic strength presumably have different influence on the activity coefficient of the reduced and oxidized species of 1,5-DHAQ (interactions with surrounding charged species) as those observed in other systems[75, 76], and thus lead to a different $E_{1/2}$.

Supplementary Note 3. The effect of driving force on the SMRT

For SMRT reactions, the driving force for reduction and oxidization of solid capacity boosting materials is governed by the potential difference between the molecule and solid capacity boosting materials.[2] Here the 1,5-DHAQ is the molecule.

For the 1,5-DHAQ, $E_{1,5-DHAQ} = E^0_{1,5-DHAQ} + \frac{RT}{2F}\ln\frac{\alpha_{1,5-DHAQ}}{\alpha_{1,5-DHAQ^{2-}}}$

For PAQS based solid capacity boosting materials (named as SM), $E_{SM} = E^0_{SM} + \frac{RT}{2F}\ln\frac{\alpha_{SM}}{\alpha_{SM^{2-}}}$

The driving force was calculated by

$$\Delta E = E_{1,5-DHAQ} - E_{SM} = E^0_{1,5-DHAQ} - E^0_{SM} + \frac{RT}{2F}\ln\frac{\alpha_{1,5-DHAQ}}{\alpha_{1,5-DHAQ^{2-}}} = \Delta E^0 + \frac{RT}{2F}\ln\frac{\alpha_{1,5-DHAQ}}{\alpha_{1,5-DHAQ^{2-}}}$$

here we used $E_{1/2}$ to instead of E^0. ΔE^0 should be as small as possible to achieve the SMRT reactions, and for the ideal situation, it should be 0. For PAQS, PAQS/CB and PAQS/CNT, the $E_{1/2}$ are -0.587, -0.588 and -0.631V (vs. SHE), which are 26, 27 and 70 mV negative than 1,5-DHAQ (-0.561 V vs. SHE), the negative ΔE makes the chemical reaction between 1,5-DHAQ^{2-} and oxidized PAQS based capacity boosting materials as rate limiting step for the SMRT based flow cell. Larger ΔE consumes more energy to overcome the energy barrier and slows down the chemical reaction rate, leading a lower utilization of capacity boosting materials. Thus, PAQS/CNT composites should have a lower utilization.

Supplementary Note 4. Head loss analysis of adding granules into the tank.

One thing needs to mention is the head loss of adding granules into the tank. As those studied in packed bed reactors, the presence of solid granules in the tank would induce a pressure drop of the fluid, which leads to additional energy loss of pump. The pressure drop is related to a few factors of the media in the storage tank, such as the porosity and tortuosity of solid granules, packing (loading) of the solid materials, flow rate, etc., which involve extensive chemical engineering optimizations. We hope we could address this in a larger-scale device in future studies.

Supplementary Note 5. Experimental evaluation of theoretical volumetric capacity of PAQS/CB granules and volumetric capacity of 1,5-DHAQ/PAQS/CB systems.

The theoretical volumetric capacity of PAQS/CB granules were calculated based on the volume change after adding the equivalent capacity of PAQS/CB granules. As shown in Supplementary Figure 39, after adding 262.5 mAh equivalent capacity of PAQS/CB granules into 20 mL 0.1 M 1,5-DHAQ/1 M KOH solution, the volume change is 0.97 mL, thus the theoretical volumetric capacity of PAQS/CB granules is calculated to be 271 Ah/L:

$$C_{granule} = \frac{262.5\ mAh}{0.97\ mL} = 271\ Ah/L$$

The volumetric capacity of 1,5-DHAQ/PAQS/CB systems were calculated based on the following equation:

$$C_{vol} = C_{granule} \times U \times P + C_{1,5-DHAQ} \times (1 - P)$$

where $C_{granule}$ (271 Ah/L) is the volumetric capacity of the PAQS/CB granules, U is the capacity utilization of PAQS, P (50%) is the packing density of solid granules, $C_{1,5-DHAQ}$ (theatrical value using 0.15 M for calculation: 8.04 Ah/L; actual value using 0.1 M for calculation: 5.36 Ah/L) is the volumetric capacity of 0.1 M 1,5-DHAQ.

Taking 50% granule loading ratio and the 33% material utilization into calculation, the actual anodic tank volumetric capacity is 47.4 Ah/L:

$$C_{vol} = 271 \times 33\% \times 50\% + 5.36 \times (1 - 50\%) = 47.4\ Ah/L$$

Taking 50% granule loading ratio and the 100% material utilization into calculation, the theoretical anodic tank volumetric capacity is 139.5 Ah/L:

$$C_{vol} = 271 \times 100\% \times 50\% + 8.04 \times (1 - 50\%) = 139.5\ Ah/L$$

Supplementary Note 6. Mechanism study of SMRT process between PAQS/CB and 1,5-DHAQ

As shown in Figure 1b, during the reduction process, the transmittance of C-O signal at 1376 cm^{-1} gradually increases, indicating the formation of 1,5-$DHAQ^{2-}$ by 1,5-DHAQ reduction. While for the same process of redox-targeting reaction of PAQS, the stretching vibration of C-O bond (1371 cm^{-1}) of PAQS solid material gradually emerges and increases (Supplementary Figure 40b), suggesting a chemical reduction process of PAQS through redox-targeting reaction between 1,5-$DHAQ^{2-}$ and PAQS forming $PAQS^{2-}$. For the oxidization process, the C-O signals of both 1,5-$DHAQ^{2-}$ and $PAQS^{2-}$ disappear, suggesting the oxidization process of 1,5-$DHAQ^{2-}$ on the electrode and chemical oxidization of $PAQS^{2-}$ through the redox-targeting reaction.

References:

1. Goulet M-A*, et al.* Extending the Lifetime of Organic Flow Batteries via Redox State Management. *Journal of the American Chemical Society* **141**, 8014-8019 (2019).

2. Zhou M*, et al.* Nernstian-Potential-Driven Redox-Targeting Reactions of Battery Materials. *Chem* **3**, 1036-1049 (2017).

3. Li Y*, et al.* Rechargeable Aqueous Polymer-Air Batteries Based on Polyanthraquinone Anode. *Chem* **5**, 2159-2170 (2019).

4. Choi W, Harada D, Oyaizu K, Nishide H. Aqueous Electrochemistry of Poly(vinylanthraquinone) for Anode-Active Materials in High-Density and Rechargeable Polymer/Air Batteries. *Journal of the American Chemical Society* **133**, 19839-19843 (2011).

5. Dražević E, Andersen AS, Wedege K, Henriksen ML, Hinge M, Bentien A. Investigation of low-cost oligoanthraquinones for alkaline, aqueous rechargeable batteries with cell potential up to 1.13 V. *Journal of Power Sources* **381**, 94-100 (2018).

6. Liang Y*, et al.* Universal quinone electrodes for long cycle life aqueous rechargeable batteries. *Nature Materials* **16**, 841-848 (2017).

7. Jing Y, Liang Y, Gheytani S, Yao Y. A Quinone Anode for Lithium-Ion Batteries in Mild Aqueous Electrolytes. *ChemSusChem* **13**, 2250-2255 (2020).

8. Kawai T, Oyaizu K, Nishide H. High-Density and Robust Charge Storage with Poly(anthraquinone-substituted norbornene) for Organic Electrode-Active Materials in Polymer–Air Secondary Batteries. *Macromolecules* **48**, 2429-2434 (2015).

9. Zhang Y*, et al.* Tetrapyridophenazine/graphene composites for aqueous hybrid flow battery anodes with long cycle life. *Carbon* **161**, 309-315 (2020).

10. Li L*, et al.* Phenazine anodes for ultralongcycle-life aqueous rechargeable batteries. *Journal of Materials Chemistry A* **8**, 26013-26022 (2020).

11. Sun T*, et al.* A phenazine anode for high-performance aqueous rechargeable batteries in a wide temperature range. *Nano Research* **13**, 676-683 (2020).

12. Dawut G, Lu Y, Miao L, Chen J. High-performance rechargeable aqueous Zn-ion batteries with a poly(benzoquinonyl sulfide) cathode. *Inorganic Chemistry Frontiers* **5**, 1391-1396 (2018).

13. Zhao Q*, et al.* High-capacity aqueous zinc batteries using sustainable quinone electrodes. *Science Advances* **4**, eaao1761.

14. Kundu D*, et al.* Organic Cathode for Aqueous Zn-Ion Batteries: Taming a Unique Phase Evolution toward Stable Electrochemical Cycling. *Chemistry of Materials* **30**, 3874-3881 (2018).

15. Alt H, Binder H, Köhling A, Sandstede G. Investigation into the use of quinone compounds-for battery cathodes. *Electrochimica Acta* **17**, 873-887 (1972).

16. Tie Z, Liu L, Deng S, Zhao D, Niu Z. Proton Insertion Chemistry of a Zinc–Organic Battery. *Angewandte Chemie International Edition* **59**, 4920-4924 (2020).

17. Li L*, et al.* Insight into the Li- and Zn-Ion Synergistic Effect for Benzoquinone-Based Anodes in Aqueous Batteries. *ACS Applied Energy Materials* **3**, 8309-8316 (2020).

18. Oka K, Furukawa S, Murao S, Oka T, Nishide H, Oyaizu K. Poly(dihydroxybenzoquinone): its high-density and robust charge storage capability in rechargeable acidic polymer–air batteries. *Chemical Communications* **56**, 4055-4058 (2020).

19. Sun T, Li Z-J, Zhi Y-F, Huang Y-J, Fan HJ, Zhang Q. Poly(2,5-Dihydroxy-1,4-Benzoquinonyl Sulfide) As an Efficient Cathode for High-Performance Aqueous Zinc–Organic Batteries. *Advanced Functional Materials* **31**, 2010049 (2021).

20. Lin Z, Shi H-Y, Lin L, Yang X, Wu W, Sun X. A high capacity small molecule quinone cathode for rechargeable aqueous zinc-organic batteries. *Nature Communications* **12**, 4424 (2021).

21. Suzuka M, Hara S, Sekiguchi T, Oyaizu K, Nishide H. Polyviologen as the charge-storage electrode of an aqueous electrolyte- and organic-based dye-sensitized solar cell. *Polymer* **68**, 353-357 (2015).

22. Liu S-M, Lin C-L, Chang T-H, Lu H-C, Hsu S-H, Ho K-C. Influence of ferrocyanide on the long-term stability of poly(butyl viologen) thin film based electrochromic devices. *Solar Energy Materials and Solar Cells* **200**, 110012 (2019).

23. Koshika K, Chikushi N, Sano N, Oyaizu K, Nishide H. A TEMPO-substituted polyacrylamide as a new cathode material: an organic rechargeable device composed of polymer electrodes and aqueous electrolyte. *Green Chemistry* **12**, 1573-1575 (2010).

24. Sano N*, et al.* Polyviologen Hydrogel with High-Rate Capability for Anodes toward an Aqueous Electrolyte-Type and Organic-Based Rechargeable Device. *ACS Applied Materials & Interfaces* **5**, 1355-1361 (2013).

25. Takahashi Y, Hayashi N, Oyaizu K, Honda K, Nishide H. Totally Organic Polymer-Based Electrochromic Cell Using TEMPO-Substituted Polynorbornene as a Counter Electrode-Active Material. *Polymer Journal* **40**, 763-767 (2008).

26. Ma T, Easley AD, Wang S, Flouda P, Lutkenhaus JL. Mixed electron-ion-water transfer in macromolecular radicals for metal-free aqueous batteries. *Cell Reports Physical Science* **2**, 100414 (2021).

27. Oka K, Murao S, Kataoka M, Nishide H, Oyaizu K. Hydrophilic Anthraquinone-Substituted Polymer: Its Environmentally Friendly Preparation and Efficient Charge/Proton-Storage Capability for Polymer–Air Secondary Batteries. *Macromolecules* **54**, 4854-4859 (2021).

28. Cao J*, et al.* A new redox-active conjugated polymer containing anthraquinone pendants as anode material for aqueous all-organic hybrid-flow battery. *Journal of Power Sources* **423**, 316-322 (2019).

29. Wang Y*, et al.* Binding Zinc Ions by Carboxyl Groups from Adjacent Molecules toward Long-Life Aqueous Zinc–Organic Batteries. *Advanced Materials* **32**, 2000338 (2020).

30. Strietzel C, Sterby M, Huang H, Strømme M, Emanuelsson R, Sjödin M. An Aqueous Conducting Redox-Polymer-Based Proton Battery that Can Withstand Rapid Constant-Voltage Charging and Sub-Zero Temperatures. *Angewandte Chemie International Edition* **59**, 9631-9638 (2020).

31. Oka K, Murao S, Kobayashi K, Nishide H, Oyaizu K. Charge- and Proton-Storage Capability of Naphthoquinone-Substituted Poly(allylamine) as Electrode-Active Material for Polymer–Air Secondary Batteries. *ACS Applied Energy Materials* **3**, 12019-12024 (2020).

32. Wang X, Xiao J, Tang W. Hydroquinone versus Pyrocatechol Pendants Twisted Conjugated Polymer Cathodes for High-Performance and Robust Aqueous Zinc-Ion Batteries. *Advanced Functional Materials* **n/a**, 2108225 (2021).

33. Ji Y*, et al.* A Phosphonate-Functionalized Quinone Redox Flow Battery at Near-Neutral pH with Record Capacity Retention Rate. *Advanced Energy Materials* **9**, 1900039 (2019).

34. Liu Y, Lu S, Chen S, Wang H, Zhang J, Xiang Y. A Sustainable Redox Flow Battery with Alizarin-Based Aqueous Organic Electrolyte. *ACS Applied Energy Materials* **2**, 2469-2474 (2019).

35. Kwabi DG*, et al.* Alkaline Quinone Flow Battery with Long Lifetime at pH 12. *Joule* **2**, 1894-1906 (2018).

36. Wu M, Bahari M, Fell EM, Gordon RG, Aziz MJ. High-performance anthraquinone with potentially low cost for aqueous redox flow batteries. *Journal of Materials Chemistry A* **9**, 26709-26716 (2021).

37. Wu M*, et al.* Extremely Stable Anthraquinone Negolytes Synthesized from Common Precursors. *Chem* **6**, 1432-1442 (2020).

38. Yang Z*, et al.* Alkaline Benzoquinone Aqueous Flow Battery for Large-Scale Storage of Electrical Energy. *Advanced Energy Materials* **8**, 1702056 (2018).

39. Lee W, Park G, Kwon Y. Alkaline aqueous organic redox flow batteries of high energy and power densities using mixed naphthoquinone derivatives. *Chemical Engineering Journal* **386**, 123985 (2020).

40. Xu J, Pang S, Wang X, Wang P, Ji Y. Ultrastable aqueous phenazine flow batteries with high capacity operated at elevated temperatures. *Joule* **5**, 2437-2449 (2021).

41. Hollas A*, et al.* A biomimetic high-capacity phenazine-based anolyte for aqueous organic redox flow batteries. *Nature Energy* **3**, 508-514 (2018).

42. Pang S, Wang X, Wang P, Ji Y. Biomimetic Amino Acid Functionalized Phenazine Flow Batteries with Long Lifetime at Near-Neutral pH. *Angewandte Chemie International Edition* **60**, 5289-5298 (2021).

43. Wang C*, et al.* Molecular Design of Fused-Ring Phenazine Derivatives for Long-Cycling Alkaline Redox Flow Batteries. *ACS Energy Letters* **5**, 411-417 (2020).

44. Jin S*, et al.* A Water-Miscible Quinone Flow Battery with High Volumetric Capacity and Energy Density. *ACS Energy Letters* **4**, 1342-1348 (2019).

45. Zhu Y*, et al.* Anthraquinone-based anode material for aqueous redox flow batteries operating in nondemanding atmosphere. *Journal of Power Sources* **501**, 229984 (2021).

46. Chang Z, Henkensmeier D, Chen R. Shifting redox potential of nitroxyl radical by introducing an imidazolium substituent and its use in aqueous flow batteries. *Journal of Power Sources* **418**, 11-16 (2019).

47. Wang H, Li D, Xu J, Wu Y, Cui Y, Chen L. An unsymmetrical two-electron viologens anolyte for salt cavern redox flow battery. *Journal of Power Sources* **492**, 229659 (2021).

48. Liu T, Wei X, Nie Z, Sprenkle V, Wang W. A Total Organic Aqueous Redox Flow Battery Employing a Low Cost and Sustainable Methyl Viologen Anolyte and 4-HO-TEMPO Catholyte. *Advanced Energy Materials* **6**, 1501449 (2016).

49. Liu B, Tang CW, Jiang H, Jia G, Zhao T. Carboxyl-Functionalized TEMPO Catholyte Enabling High-Cycling-Stability and High-Energy-Density Aqueous Organic Redox Flow Batteries. *ACS Sustainable Chemistry & Engineering* **9**, 6258-6265 (2021).

50. Janoschka T, Martin N, Hager MD, Schubert US. An Aqueous Redox-Flow Battery with High Capacity and Power: The TEMPTMA/MV System. *Angewandte Chemie International Edition* **55**, 14427-14430 (2016).

51. Chang Z, Henkensmeier D, Chen R. One-Step Cationic Grafting of 4-Hydroxy-TEMPO and its Application in a Hybrid Redox Flow Battery with a Crosslinked PBI Membrane. *ChemSusChem* **10**, 3193-3197 (2017).

52. Hagemann T*, et al.* (2,2,6,6-Tetramethylpiperidin-1-yl)oxyl-Containing Zwitterionic Polymer as Catholyte Species for High-Capacity Aqueous Polymer Redox Flow Batteries. *Chemistry of Materials* **31**, 7987-7999 (2019).

53. Janoschka T*, et al.* An aqueous, polymer-based redox-flow battery using non-corrosive, safe, and low-cost materials. *Nature* **527**, 78-81 (2015).

54. Liu Y*, et al.* A Long-Lifetime All-Organic Aqueous Flow Battery Utilizing TMAP-TEMPO Radical. *Chem* **5**, 1861-1870 (2019).

55. Beh ES, De Porcellinis D, Gracia RL, Xia KT, Gordon RG, Aziz MJ. A Neutral pH Aqueous Organic–Organometallic Redox Flow Battery with Extremely High Capacity Retention. *ACS Energy Letters* **2**, 639-644 (2017).

56. DeBruler C, Hu B, Moss J, Luo J, Liu TL. A Sulfonate-Functionalized Viologen Enabling Neutral Cation Exchange, Aqueous Organic Redox Flow Batteries toward Renewable Energy Storage. *ACS Energy Letters* **3**, 663-668 (2018).

57. Hu S*, et al.* Phenylene-Bridged Bispyridinium with High Capacity and Stability for Aqueous Flow Batteries. *Advanced Materials* **33**, 2005839 (2021).

58. Jin S*, et al.* Near Neutral pH Redox Flow Battery with Low Permeability and Long-Lifetime Phosphonated Viologen Active Species. *Advanced Energy Materials* **10**, 2000100 (2020).

59. Luo J, Hu B, Debruler C, Liu TL. A π-Conjugation Extended Viologen as a Two-Electron Storage Anolyte for Total Organic Aqueous Redox Flow Batteries. *Angewandte Chemie International Edition* **57**, 231-235 (2018).

60. Li H, Fan H, Hu B, Hu L, Chang G, Song J. Spatial Structure Regulation: A Rod-Shaped Viologen Enables Long Lifetime in Aqueous Redox Flow Batteries. *Angewandte Chemie International Edition* **n/a**, (2021).

61. DeBruler C*, et al.* Designer Two-Electron Storage Viologen Anolyte Materials for Neutral Aqueous Organic Redox Flow Batteries. *Chem* **3**, 961-978 (2017).

62. Huskinson B*, et al.* A metal-free organic–inorganic aqueous flow battery. *Nature* **505**, 195-198 (2014).

63. Gerhardt MR*, et al.* Anthraquinone Derivatives in Aqueous Flow Batteries. *Advanced Energy Materials* **7**, 1601488 (2017).

64. Park M*, et al.* A High Voltage Aqueous Zinc–Organic Hybrid Flow Battery. *Advanced Energy Materials* **9**, 1900694 (2019).

65. Yang B, Hoober-Burkhardt L, Krishnamoorthy S, Murali A, Prakash GKS, Narayanan SR. High-Performance Aqueous Organic Flow Battery with Quinone-Based Redox Couples at Both Electrodes. *Journal of The Electrochemical Society* **163**, A1442-A1449 (2016).

66. Sun P*, et al.* 110th Anniversary: Unleashing the Full Potential of Quinones for High Performance Aqueous Organic Flow Battery. *Industrial & Engineering Chemistry Research* **58**, 3994-3999 (2019).

67. Lin K*, et al.* Alkaline quinone flow battery. *Science* **349**, 1529 (2015).

68. Tong L*, et al.* Molecular Engineering of an Alkaline Naphthoquinone Flow Battery. *ACS Energy Letters* **4**, 1880-1887 (2019).

69. Wang C, *et al.* High-Performance Alkaline Organic Redox Flow Batteries Based on 2-Hydroxy-3-carboxy-1,4-naphthoquinone. *ACS Energy Letters* **3**, 2404-2409 (2018).

70. Lin K, *et al.* A redox-flow battery with an alloxazine-based organic electrolyte. *Nature Energy* **1**, 16102 (2016).

71. Orita A, Verde MG, Sakai M, Meng YS. A biomimetic redox flow battery based on flavin mononucleotide. *Nature Communications* **7**, 13230 (2016).

72. Huang J, *et al.* Spatially Constrained Organic Diquat Anolyte for Stable Aqueous Flow Batteries. *ACS Energy Letters* **3**, 2533-2538 (2018).

73. Hu B, Tang Y, Luo J, Grove G, Guo Y, Liu TL. Improved radical stability of viologen anolytes in aqueous organic redox flow batteries. *Chemical Communications* **54**, 6871-6874 (2018).

74. Yang L, *et al.* Unveiling the high-activity origin of single-atom iron catalysts for oxygen reduction reaction. *Proceedings of the National Academy of Sciences* **115**, 6626-6631 (2018).

75. DeFord DD, Andersen DL. The Effect of Ionic Strength on Polarographic Half-wave Potentials1. *Journal of the American Chemical Society* **72**, 3918-3920 (1950).

76. Vanderborgh NE, Sellers DE. The Influence of Ionic Strength on Polarographic Half-Wave Potentials. II. The Investigation of the Reduction of Zinc and Nickel Nitrates and the Effect of Mixed Uni-univalent Inert Electrolytes on the Reduction of Cadmium Nitrate. *Journal of the American Chemical Society* **86**, 2790-2792 (1964).
